# Identification of Nsp15 inhibitors restoring interferon-dependent antiviral activity against SARS-CoV-2

**DOI:** 10.1016/j.antiviral.2026.106443

**Published:** 2026-05-22

**Authors:** Stefania Maloccu, Yuka Otsuka, Elizabeth Molchan, Michelle Foti, Annalaura Paulis, Jyoti Vishwakarma, Paolo Malune, Filippo Cottiglia, Angela Corona, Louis Scampavia, Timothy P. Spicer, Reuben S. Harris, Enzo Tramontano, Elisa Fanunza

**Affiliations:** a Department of Life and Environmental Science, University of Cagliari, Cagliari, Italy; b Department of Molecular Medicine, The Herbert Wertheim UF Scripps Institute for Biomedical Innovation & Technology, Jupiter, FL, USA; c Department of Biochemistry and Structural Biology, University of Texas Health San Antonio, San Antonio, TX, USA; d Howard Hughes Medical Institute, University of Texas Health San Antonio, San Antonio, TX, USA

**Keywords:** SARS-CoV-2, Endoribonuclease, Nsp15, Interferon system, Reactive blue 2, HTS screening, Drug development

## Abstract

The endoribonuclease Nsp15 is essential for coronavirus pathogenesis and evasion of host defenses and is therefore a promising drug target. We determined optimal parameters for an endoribonuclease FRET biochemical assay and use it for a high-throughput drug screen. Measurement of Z′-factor confirmed robust assay performance (Z’ = 0.7– 0.9). We screened a commercially available library (LOPAC 1280) and identified three molecules able to inhibit the catalytic endonuclease activity of Nsp15 in the low micromolar range. Among them, a promising hit compound, Reactive Blue 2 showed also anti-SARS-CoV-2 activity with EC_50_ value of 2 μM, and low cytopathic effect in multiple cell types (CC_50_ > 28 μM). More importantly, SARS-CoV-2 replication was inhibited in cells with an intact IFN system, but not in IFN-deficient cell lines.

## 1. Introduction

SARS-CoV-2 continues to pose a global threat, with more than 778 million of total cases reported to WHO and 7.1 million deaths globally. Despite successful efforts to develop vaccines and antivirals, the virus continues to circulate, accounting around 300 deaths in the last month (COVID-19 Deaths | WHO COVID-19 Dashboard, n.d.). Current antiviral development for SARS-CoV-2 has largely focused on conserved viral enzymes such as the RNA-dependent RNA polymerase (RdRp) and viral proteases(Cannalire et al., 2022; Malune et al., 2025; Otsuka et al., 2024). However, reliance on these targets alone may be insufficient to address emerging variants and future coronavirus threats. There is a critical need to expand the antiviral arsenal by exploring alternative and underexploited viral targets.

One such promising target is the viral uridylate-specific endoribonuclease, Nsp15. Nsp15 is a conserved non-structural protein across the Nidovirales order and plays essential roles in viral immune evasion and RNA processing(Deng and Baker, 2018). As a major interferon (IFN) antagonist, coronaviruses Nsp15 suppresses host antiviral responses through multiple mechanisms i) by cleaving RNA, it is assumed to prevent detection of the viral RNA intermediates by host pattern recognition receptors such as MDA5; ii) it physically interacts with host immune signaling proteins: it binds TANK-binding kinase 1 (TBK1), disrupting TBK1-IRF3 interaction and thereby blocking IRF3 phosphorylation and downstream signaling; iii) it leads to karyopherin α1 (KPNA1) degradation, impeding nuclear translocation of activated IRF3 (Hayn et al., 2021; Otter et al., 2024; Zhang et al., 2023).

The endoribonuclease activity of Nsp15 resides in its C-terminal EndoU domain, which selectively cleaves uridylate-rich single-stranded (ssRNA) and double-stranded RNA (dsRNA) regions with a strong preference for unpaired or bulged uridines (Wright et al., 2025). The active site contains a conserved catalytic triad of H235, H250, and K290, which facilitate acid-base catalysis similar to RNase A. These residues coordinate Mn^2+^ or other divalent cations for optimal activity (Frazier et al., 2021). This enzymatic activity not only facilitates immune evasion but is also implicated in viral RNA maturation and the efficient function of the replication-transcription complex (RTC), where Nsp15 colocalizes with other viral proteins (Gong et al., 2025; Saramago et al., 2022).

The critical role of Nsp15 in pathogenesis has been demonstrated in various CoV models. For example, EndoU-deficient Mouse Hepatitis Virus (MHV) mutants show attenuated virulence, reduced viral titers, and diminished tissue pathology in vivo. Similarly, Porcine Epidemic Diarrhea Virus (PEDV) exploits Nsp15 to suppress host IFN responses and cause lethal disease in piglets. In SARS-CoV-2, catalytic inactivation of Nsp15 leads to heightened IFN signaling and impaired viral replication, highlighting the endoribonuclease as an important virulence factor and an attractive target for therapeutic intervention(Caobi et al., 2025; Chi et al., 2025; Deng and Baker, 2018; Otter et al., 2024).

Despite its potential, Nsp15 remains an underexplored antiviral target. Nowadays, a few compounds have been reported to inhibit its enzymatic activity in the micromolar range. However, these candidates are generally limited by none or poor antiviral activity in cell-based assays (Chen et al., 2023; Hurst et al., 2021; Kim et al., 2021; Van Loy et al., 2024).

In search for Nsp15 inhibitors, we optimized a high-throughput biochemical assay for the endonuclease activity and identified a novel small-molecule inhibitor of Nsp15. This compound exhibits inhibition of SARS-CoV-2 Nsp15 endoribonuclease activity (IC_50_ = 13.71 μM) and robust antiviral activity in SARS-CoV-2-infected cells (EC_50_ = 2 μM). Notably, antiviral efficacy was observed only in cells with an intact interferon system, but not in IFN-deficient models, suggesting an IFN-dependent mechanism of action. This specificity provides both a mechanistic validation of Nsp15 role in immune suppression and a therapeutic advantage in preserving host innate immunity.

## 2. Materials and methods

### 2.1. Cells and viruses

Cells used in viral replication assays were HEK293TN-ACE2 cells kindly provided by Prof. Raffaele de Francesco (INMI, Italy) and generated as previously reported (Andreano et al., 2023), and Vero E6 GFP cells (kindly provided by Janssen Pharmaceutical). HEK293TN-ACE2 were maintained in Dulbecco’s modified Eagle’s medium (DMEM; Gibco) supplemented with 10% v/v fetal beef serum heat inactivated (FBS-HI; Gibco), and 1x Pen-strep (Euroclone) and kept under 5% CO_2_ on 37 °C. Vero E6 GFP cells were maintained in DMEM (Gibco) supplemented with 10% v/v FBS-HI (Gibco), 0.075% Sodium Bicarbonate (7.5% solution, Gibco) and 1x Pen-strep (Euroclone) and kept under 5% CO_2_ on 37 °C.

Cells used for qRT-PCR and co-immunoprecipitation were HEK293T (ATCC), maintained in DMEM (Gibco) supplemented with 10% v/v FBS (Gibco), and 1x Pen-strep (Euroclone) and kept under 5% CO_2_ on 37 °C.

For antiviral replication assays, the virus used was a SARS-CoV-2 strain BetaCov/Belgium/GHB-03021/2020, provided by KU Leuven and propagated in Vero E6 GFP cells. All experiments involving SARS-CoV-2 were conducted in the Biosafety Level 3 Laboratory at the University of Cagliari.

Sendai Virus (SeV) used for stimulation of IFNβ production cascade was purchased from ATCC (VR-907), and propagated in Vero E6 GFP cells, using DMEM (Gibco) supplemented with 5% v/v FBS-HI (Gibco), 1.5 mg/mL Trypsin (Euroclone) and 1x Pen-strep (Euroclone) and kept under 5% CO_2_ on 37 °C. Viral stocks titer was calculated with the 50% tissue culture infection dose (TCID_50_) method.

### 2.2. Expression and purification of Recombinant Nsp15 proteins

Recombinant Nsp15 proteins from SARS-CoV-2 (Omicron variant) and MERS-CoV were expressed in *E*. *coli* for subsequent biochemical characterization. The gene encoding Nsp15 from the Omicron variant and MERS-CoV were codon-optimized for bacterial expression and synthesized by GenScript (Piscataway, NJ, USA). These sequences, available in Supplementary Table 1, were cloned using restriction enzymes Nhe-I and Not-I into a pET28b (+) with a N-terminal 6×His, or SUMO-6×His tags. Plasmids were transformed into *E. coli* BL21(DE3) Gold competent cells (Agilent Technologies). Single colonies were used to inoculate starter cultures grown overnight at 37 °C in Luria-Bertani (LB) medium supplemented with 50 μg/ml kanamycin. The next day, cultures were diluted 1:100 into fresh LB-ampicillin medium and grown at 37 °C with shaking until reaching an optical density (OD_600_) of 0.6. Cultures were cooled at 17 °C for 1h, and protein expression was induced by the addition of isopropyl β-D-1-thiogalactopyranoside (IPTG) to a final concentration of 0.5 mM or 1.0 mM. Induced cultures were incubated at 18 °C, 30 °C, or 37 °C for 16 h. Optimal expression was observed at 18 °C with 0.5 mM IPTG, and these conditions were used for all subsequent protein preparations. The bacterial pellets were collected by centrifugation and stored at −80 °C. The recombinant proteins purification was performed by affinity chromatography using a Ni^2+^-Sepharose resin and size-exclusion chromatography (Superdex 200 Increase 10/300 GL) using Akta Pure 25M (Cytiva). The obtained protein was concentrated using Amicon^®^ Ultra centrifugal filters 30 kDa (Millipore Sigma). Protein concentration was determined with a nanodrop, and purity was assessed by SDS-PAGE.

### 2.3. SDS-PAGE, western blot and native PAGE analysis

SDS-PAGE was performed using 12% polyacrylamide gels under reducing conditions. Gels were stained with Coomassie Brilliant Blue R-250. As shown in, His-tagged Omicron and MERS-CoV Nsp15 migrated at ~41 kDa, consistent with their expected molecular weight. The SUMO-His-tagged Omicron Nsp15 migrated at ~55 kDa (Supplementary Figure 1A).

Western blotting was carried out as previously described(Kuo et al., 2021). Proteins were transferred onto PVDF membranes and blocked with 5% non-fat dry milk in TBST (20 mM Tris-HCl, pH 7.5, 150 mM NaCl, 0.1% Tween-20) for 1 h at room temperature. Membranes were probed with a rabbit polyclonal antibody specific to SARS-CoV-2 Nsp15 (1:1000 dilution; Invitrogen) overnight at 4 °C, followed by HRP-conjugated anti-rabbit secondary antibody (1:10,000). Detection was performed using ECL substrate (Thermo Fisher Scientific). Specific immunoreactive bands were detected corresponding to both His-tagged and SUMO-His-tagged Omicron Nsp15, confirming the identity of the purified proteins (Supplementary Figure 1B).

Native page analysis was performed to check for the oligomerization state of SARS-CoV-2 Nsp15. 1 μg of Nsp15 purified protein was loaded in a 3–12% Native Page gel (Thermo Fisher Scientific) using the NativeMark^™^ Unstained Protein Standard (Code LC0725 Thermo Fisher Scientific) as molecular weight indicator. The gel was stained with Simply Blue Safe stain (Thermo Fisher Scientific) and visualized with a ChemiDoc Imager (BioRad). As shown in Supplementary Figure 1C, Nsp15 migrates as a single, discrete band at a position corresponding to approximately 250 kDa, which matches the predicted size of the hexameric complex. The absence of bands at the monomeric weights (41 KDa) suggests that the hexamer is the predominant and stable form under the conditions tested.

### 2.4. FRET endoribonuclease assay

Real-time monitoring of Nsp15-mediated cleavage was performed as previously described using a FRET biochemical assay(Frazier et al., 2021). Optimization was performed to adapt it to large scale drug screening in 384-wfp, using a final reaction volume of 40 μL, and 1536-wfp using a final reaction volume of 8 μL. Briefly, 6-mer RNA substrates were dual-labeled with a 5′-fluorescein (5′−6-FAM) donor and a 3′-TAMRA quencher (5′−6-FAM-AAAUAA-3′-TAMRA). TAMRA suppresses FAM fluorescence, allowing cleavage activity to be tracked by an increase in FI signal upon substrate cleavage. Reactions were set up with 1.5 μM (or 0.75 μM) RNA substrate and 30 nM (or 7.5 nM) Nsp15 in the 384-wpf (or 1536-wpf) in RNA cleavage buffer (20 mM HEPES, pH 7.0, 75 mM NaCl, 5 mM MgCl_2_, 5 mM DTT, 0.05 mg/mL BSA) and incubated at 25 °C for 60 min. Fluorescence was recorded every 2.5 min using a Nivo 5 plate reader (PerkinElmer) with excitation at 485 nm and emission at 520 nm. For LOPAC screening, plates were incubated 30 min at room temperature and fluorescent was measured using PHERAstar (BMG). Raw assay data was imported into UF-Scripps’ corporate database (Baillargeon et al., 2019) and subsequently analyzed using Symyx software. Activity of each compound was calculated on a per-plate basis using the following equation:

$$\mathrm{Percent}\;\mathrm{Response}\;\mathrm{of}\;\mathrm{compound}\;=100\;\;\times \left(\frac{\mathrm{Test}\;\mathrm{Well}\;-\;\mathrm{Median}\;\mathrm{Data}\;\mathrm{Wells}}{\mathrm{Median}\;\mathrm{High}\;\mathrm{Control}\;-\;\mathrm{Median}\;\mathrm{Data}\;\mathrm{Wells}}\right)$$

Where the “High Control” represents wells containing Nsp15 + negative substrate + DMSO. “Low Control” represents wells containing Nsp15 + positive substrate + DMSO and “Data Wells” contain the same including test compounds. The Z′ and signal-to-background ratio (S:B) for this assay is calculated using the High Control and Low Control wells.

The HIV-1 RT-associated RNase H expressed and purified as previously reported (Corona et al., 2020) was used as additional internal negative control at concentration range of 10–750 nM.

Compounds, solubilized in DMSO, were tested at different concentrations. The IC_50_ values were calculated using GraphPad Prism software Version 9.1.2. Test compound results were normalized relative to respective controls. Dose-response curves were fitted to a non-linear regression of (log10) dose vs normalized response-variable slope. Results are reported as average and standard deviation of three independent experiments performed in triplicate.

### 2.5. Endoribonuclease page assay

Nsp15 at decreasing concentration (622 nM-207 nM-70 nM-23 nM-7,5 nM-2,56 nM-0,86 nM - 0,2 nM) was incubate in reaction buffer [20 mM Hepes (pH 7.0), 75 mM NaCl, 5 mM MgCl_2_, 5 mM DTT, 0.05 mg/ml BSA], with 0.75 μM substrate (5′−6-FAM-AAAUAA-3′-TAMRA) for 30′ at room temperature. Decreasing concentrations (30–1.1 μM) of Reactive Blue 2 were added at the reaction. Then, reaction was blocked with 2X volume of stopped buffer (formamide with 4% v/v EDTA 0.5 M, pH 8.0). Samples were denatured at 95 °C for 5′ and resolved by electrophoresis 15% Urea-page (7M urea, 19:1 acrylamide/bis-acrylamide). Gel was scanned using a ChemiDoc Imager (Bio-Rad).

### 2.6. Cytotoxicity assay

The cellular toxicity of the compounds in VeroE6-GFP cells was evaluated by GFP constitutive expressed fluorescence. Cells were seeded at 10,000 cells/well in 96-well black plates. The following day, cells were incubated, with or without compounds, in presence of 2 μM P-gp inhibitor CP-100356 (a P-glycoprotein pump inhibitor). Briefly, VeroE6-GFP monolayers were incubated with different concentrations of compounds starting from the highest concentration of 100 μM, in the presence of 2 μM CP-100356 for 72 h. Compounds were dissolved in 0.1% dimethyl sulfoxide (DMSO). After 72 h post-treatment media was removed and the total well GFP fluorescence was measured using Victor NIVO (PerkinElmer) with 485/535 nm excitation wavelength.

The cytopathic effect was quantified using the GFP redout. For each experimental plate, the GFP signal of untreated (U cell-only) control wells were used to represent 100% viable cells, the difference in GFP signal between U and cell free wells (N) served as a control representing maximal cell viability. The treated samples were normalized as % of cell viability using the following formula:

$$\%\mathrm{of}\;\mathrm{viability}\;=\frac{\;\mathrm{sample}\;-\;(\;\mathrm{mean}\;\mathrm{of}\;U)}{\left(\mathrm{mean}\;\mathrm{of}\;N\;-\;\mathrm{mean}\;\mathrm{of}\;U\right)}*100$$

The antiviral activity of test compounds was expressed as EC_50_, concentration of compound able to determine a 50% reduction of the CPE, and was determined via non-linear regression using GraphPad Prism Version 9.1.2 with the function dose-response curve, non-linear regression of (log_10_) dose vs normalized response (variable slope).The results are representative of two biological replicates in triplicate.

HEK293TN-ACE2 cells were seeded into clear 96-well plates at a concentration of 1 × 10^5^ cells/mL, 100 μL per well). On day 2, cells were incubated with the selected compounds at different concentrations. Compounds were dissolved in 0.1% dimethyl sulfoxide (DMSO). After 72 h post-treatment (depending on the cell line and the specific antiviral assay), 20 μl of 3-(4,5-dimethylthiazol-2-yl)-2,5-diphenyl-2H-tetrazolium bromide (MTT) dissolved in PBS at 7.5 mg/ml, were added to each well and the cells were incubated at 37 °C with 5% CO_2_ for 1 h. Then the supernatant was removed, and cells were lysed with 100 μl/well of lysis buffer (100% 2-Propanol, 4% Triton-X-100, 0,4% Hydrogen Chloride) until the formazan crystals were completely dissolved, then the absorbance was read at 570 nm with a plate reader (Victor Nivo, PerkinElmer).

The cytopathic effect was quantified using the MTT redout. For each experimental plate, the MTT signal of untreated (U: cell-only) control wells were used to represent 100% viable cells, the difference in MTT signal between U and cell free wells (N) served as a control representing maximal cell viability. The treated samples were normalized as % of cell viability using the following formula:

$$\%\;\mathrm{of}\;\mathrm{viability}\;=\frac{\;\mathrm{sample}\;-\;(\mathrm{mean}\;\mathrm{of}\;U)}{\left(\mathrm{mean}\;\mathrm{of}\;N\;-\;\mathrm{mean}\;\mathrm{of}\;U\right)}*100$$

The antiviral activity of test compounds was expressed as EC_50_, concentration of compound able to determine a 50% reduction of the CPE, and was determined via non-linear regression using GraphPad Prism Version 9.1.2 with the function dose-response curve, non-linear regression of (log_10_) dose vs normalized response (variable slope).The results are representative of two biological replicates in triplicate.

### 2.7. Viral replication assays

#### - SARS-CoV-2 Viral Replication Inhibition Assay in HEK293TN-ACE2 Cells

HEK293TN-ACE2 cells were seeded into clear 96-well plates at a concentration of 1 × 10^5^ cells/mL, 100 μL per well, to achieve 90% confluence 24 h after seeding.

The following day, cells were infected with a multiplicity of infection (MOI) of 0.75 (15000 PFU/well), as the previously determined virus dose capable of inducing 80% cell death at 72 h post-infection, and treated either with the test compounds or with 0.1% DMSO (untreated control). GC376 was used as a positive control for viral replication inhibition (Vuong et al., 2021). At 72 h post-infection, cells were treated with a 7.5 mg/mL solution of 3-(4,5-dimethylthiazol-2-yl)-2,5-diphenyltetrazolium bromide (MTT) and incubated for 1 h at 37 °C in a 5% CO_2_ atmosphere. The supernatant was then removed, and the cells were lysed with a solution containing isopropanol, 0.4% HCl, and 6% Triton X-100. The plate was shaken at 200 rpm until complete dissolution of the formazan crystals. Absorbance was then measured using a Cytation-5 plate reader at 570 nm.

Viral Induced cytopathic effect (CPE) was quantified using MTT redout. For each experimental plate, the MTT signal of uninfected untreated (UU: cell-only) control wells were used to represent 100% viable cells, the difference in MTT signal between UU and virus-infected untreated (VU) wells served as a control representing maximal CPE. The treated samples were normalized as % of CPE using the following formula:

$$\%\;\mathrm{of}\;CPE=\frac{\;\mathrm{sample}\;-\;(\mathrm{mean}\;\mathrm{of}\;VU)}{\left(\mathrm{mean}\;\mathrm{of}\;UU\;-\;\mathrm{mean}\;\mathrm{of}\;VU\right)}*100$$

The antiviral activity of test compounds was expressed as EC_50_, concentration able to determine a 50% reduction of the CPE, and was determined via non-linear regression using GraphPad Prism Version 9.1.2 with the function dose-response curve, non-linear regression of (log_10_) dose vs normalized response (variable slope).The results are representative of two biological replicates in triplicate.

#### - SARS-CoV-2 Viral Replication Inhibition Assay in Vero E6 GFP Cells

Vero E6 GFP cells were seeded into black 96-well plates at a concentration of 1 × 10^5^ cells/mL, 100 μL per well. The following day, cells were infected with SARS-CoV-2 a MOI of 0.01 in the presence of 2 μM CP-100356 (a P-glycoprotein pump inhibitor) and treated either with the test compounds or with 0.1% DMSO (untreated control). GC376 was used as a positive control for viral replication inhibition. The possible cytotoxic effect exerted by the compound was assessed on the same plate in treated uninfected samples. After 72 h post-infection, the supernatant was removed, and total fluorescence was measured using a Victor Nivo5 plate reader (PerkinElmer) at 485/535 nm (excitation/emission). Viral replication inhibition was calculated as the percentage of cytopathic effect reduction compared to the untreated, infected control. In parallel, the cytotoxic effect of the compounds was evaluated in treated but uninfected cells, calculating cell viability as a percentage relative to the uninfected control. Viral Induced cytopathic effect (CPE) was quantified using GFP redout. For each experimental plate, the GFP signal of uninfected untreated (UU: cell-only) control wells were used to represent 100% viable cells, the difference in GFP signal between UU and virus-infected untreated (VU) wells served as a control representing maximal CPE. The treated samples were normalized as % of CPE using the following formula:

$$\%\mathrm{of}\;CPE=\frac{\;\mathrm{sample}\;-\;(\;\mathrm{mean}\;\mathrm{of}\;VU)}{\left(\mathrm{mean}\;\mathrm{of}\;UU\;-\;\mathrm{mean}\;\mathrm{of}\;VU\right)}*100$$

The antiviral activity of test compounds was expressed as EC_50_, concentration of compound able to determine a 50% reduction of the CPE, and was determined via non-linear regression using GraphPad Prism Version 9.1.2 with the function dose-response curve, non-linear regression of (log_10_) dose vs normalized response (variable slope).The results are representative of two biological replicates in triplicate.

### 2.8. Surface plasmon resonance (SPR) and microscale thermophoresis (MST) assays

To determine the binding affinity between SARS-CoV-2 Nsp15 protein and compound Reactive Blue 2 an SPR-based assay was performed in a Biacore^™^ X100 SPR system (Cytiva).

Briefly, Nsp15 protein (50 μg/ml in sodium acetate pH 4.5 buffer) was coupled to a CM5 chip, following the amine conjugation protocol in HBS-EP + 1X running buffer (Cytiva). The compound dissolved in DMSO was serially diluted in running buffer (50 mM Tris-HCl pH 7.5, 500 mM NaCl, 5 mM MnCl_2_, 0.05% v/v Tween20, 5% DMSO) as previously described (Van Loy et al., 2025a) and injected onto the Nsp15-coupled and reference channels, with binding and dissociation durations of 80 s and 120 s, respectively. Binding data was recorded and analyzed using the Biacore^™^ X100 Control and Evaluation Software (Cytiva).

MST was used to determine whether Reactive Blue 2 was able to bind Nsp15 or our substrate RNA. We used a reaction mixture of 20 mM Hepes pH 7.0, 75 mM NaCl, 5 mM MgCl_2_, 0.05% Tween and 0,05 mg/ml BSA. Since the compound is fluorescent, a reverse MST experiment was performed using its intrinsic fluorescence. The optimal concentration of Reactive Blue 2 was determined to be 10 μM. Based on this, a binding check was carried out using 10 μM Nsp15 (Supplementary Fig. 9A) and 50 μM RNA substrate (Supplementary Fig. 9B).

### 2.9. RT-qPCR for quantification of IFNβ expression

For IFNβ cascade activation, cells were transfected with an empty vector pcDNA3.1 or pcDNA3.1-V5-Nsp15. A day post-transfection, cells were infected with SeV (MOI = 0.1) for 24 h, and treated or not with Reactive Blue 2 (10 μM). Total RNA was isolated from transfected cells using TRIzol reagent (Invitrogen). cDNA synthesis and amplification were carried out with the Luna Universal One-Step RT-qPCR Kit (New England BioLabs). Primers used were: glyceraldehyde-3-phosphate dehydrogenase (GAPDH) Forward 5′-GAGTCAACGGATTTTGGTCGT-3′, GAPDH Reverse 5′-TTGATTTTGGAGGGATCTCG-3′, IFN-beta Forward 5′-CTTGGATTCCTACAAAGAAGCAGC-3′, IFN-beta Reverse 5′-TCCTCCTTCTGGAACTGCTGCA-3’, as previously described (Corona et al., 2022). mRNA IFNβ levels were normalized to GAPDH expression. Data are presented as the percentage of transcript abundance in treated samples relative to untreated controls. All RT-qPCR assays were conducted in triplicate in a CFX-96 Real-Time system (Bio-Rad).

### 2.10. Co-immunoprecipitation

HEK293T cells were transfected with empty vector or expression plasmids for V5-tagged Nsp15 and FLAG-tagged KPNA1 for 24 h, following treatment with Reactive Blue 2 (10 μM). Ebola VP24 was used as protein control for co-immunoprecipitation with KPNA1 as previously reported (Fanunza et al., 2020). Cells were lysed in MCLB Buffer containing 50 mM Tris pH 7.5, 300 nM NaCl, 0.5% NP-40, 1 mM DTT and cOmplete protease inhibitor cocktail (Roche). Cellular lysates were incubated with magnetic beads anti-FLAG M2 (Sigma) overnight at 4 °C. Elutions were performed adding to the samples FLAG peptide (Sigma). Elutions and whole cell lysates were analyzed by Western blotting. Membranes were probed with anti-FLAG M2 (Sigma-Aldrich, 1:2000), anti-V5 (Thermo Fisher Scientific; 1:2000), and anti-GAPDH (Cell Signaling; 1:1000). Chemidoc MP imaging system (Bio-Rad) was used to capture WB images.

### 2.11. Statistical analysis and graphic visualization

**Figure 1.**
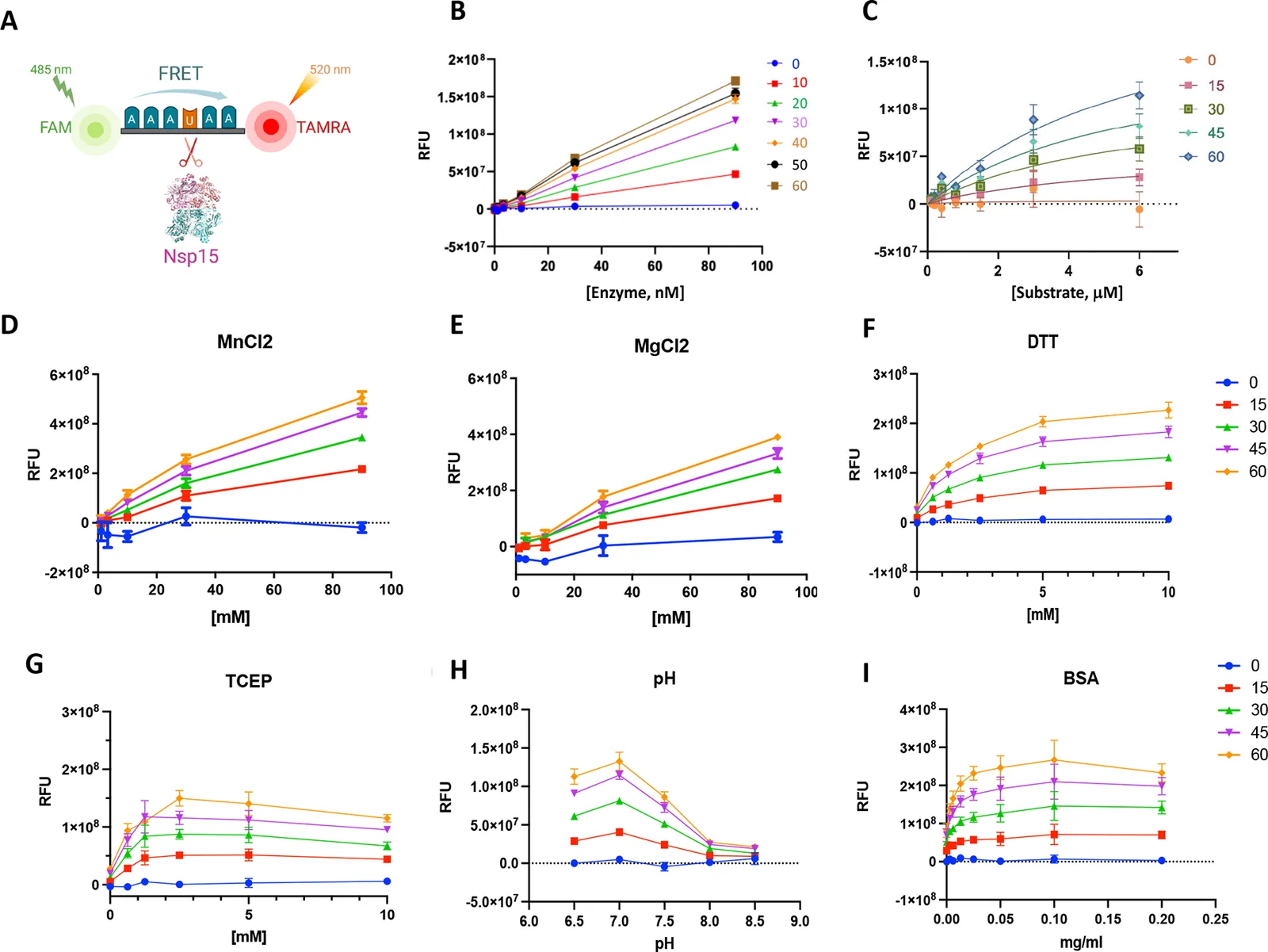
Measurements of Nsp15 endonuclease activity using the FRET-based assay. **(A)** Schematic representation of FRET endonuclease assay, created in BioRender. Harris, R. (2026) https://BioRender.com/yuk8sjw; **(B)** Dose-response curve for Nsp15; **(C)** Dose-response curve for Substrate; Evaluation of metal ions, MnCl_2_ **(D)** or MgCl_2_ **(E)**, reducing agents, DTT **(F)** or TCEP **(G)**, pH **(H)** and BSA **(I)** on Nsp15 EndoU activity. Different line colors indicate different time points (minutes), relative fluorescence units (RFU). Data are expressed as the means ± standard deviations (SDs) from at least three independent experiments.

Statistical analysis and graphic visualization were performed using GraphPad Prism software 9.1.2 (GraphPad Software, Inc.). Fig. 1A was created with Biorender (https://www.biorender.com).

## 3. Results

### 3.1. Optimization of a FRET-based biochemical assay for Nsp15 endoribonuclease activity

To assess Nsp15 endoribonuclease activity *in vitro*, we optimized a FRET-based biochemical assay for high-throughput screening applications (Frazier et al., 2021). The assay employs a single-stranded RNA substrate containing a central uridine residue. Upon cleavage by Nsp15, the 5′−6-FAM fluorophore is separated from the 3′-TAMRA quencher, resulting in an increase in fluorescence signal (Fig. 1A).

Upon assessing expression and oligomerization state of purified recombinant Omicron His-tagged Nsp15 (Supplementary Fig. 1), we performed a dose-response curve using at increasing concentrations of enzyme and monitored fluorescence over time, expressed in relative fluorescence units (RFU) (Fig. 1B). Enzyme activity increased proportionally with Nsp15 concentration, and 30 nM was selected for all subsequent assays as it was within the linear range of the kinetic curve. Next, we optimized substrate concentration by titrating the FRET-labeled RNA and measuring activity at a fixed enzyme concentration. A concentration of 2 μM substrate provided a robust and reproducible signal (Fig. 1C) and was used in follow-up experiments. Under these conditions (30 nM enzyme, 2 μM substrate), fluorescence reached ~5 × 10^7^ RFU after 60 min, offering an excellent dynamic range.

Previous studies reported a requirement for Mn^2+^ ions to support optimal Nsp15 activity(Saramago et al., 2022). We confirmed that 5 mM MnCl_2_ enhanced RNA cleavage by Omicron His-Nsp15 (Fig. 1D). However, considering the low physiological concentrations of Mn^2+^ in cells, and the risk of Mn^2+^ promoting false positives during compound screening, we tested Mg^2+^ as an alternative. Notably, 5 mM MgCl_2_ supported Nsp15 activity to a similar extent as Mn^2+^ (Fig. 1E), making it a suitable and more physiologically relevant cofactor for all subsequent assays.

We further refined assay conditions by testing the impact of reducing agents on enzyme performance. Dithiothreitol (DTT) promoted higher Nsp15 activity compared to TCEP (Fig. 1F and G). We also evaluated buffer pH and found that activity was optimal at pH 7.0 (Fig. 1H). The addition of 0.1% bovine serum albumin (BSA) was used to minimize background signal of nonspecific adsorption of proteins in biochemical assays (Fig. 1I).

### 3.2. Evaluation of enzymatic kinetics

**Figure 2.**
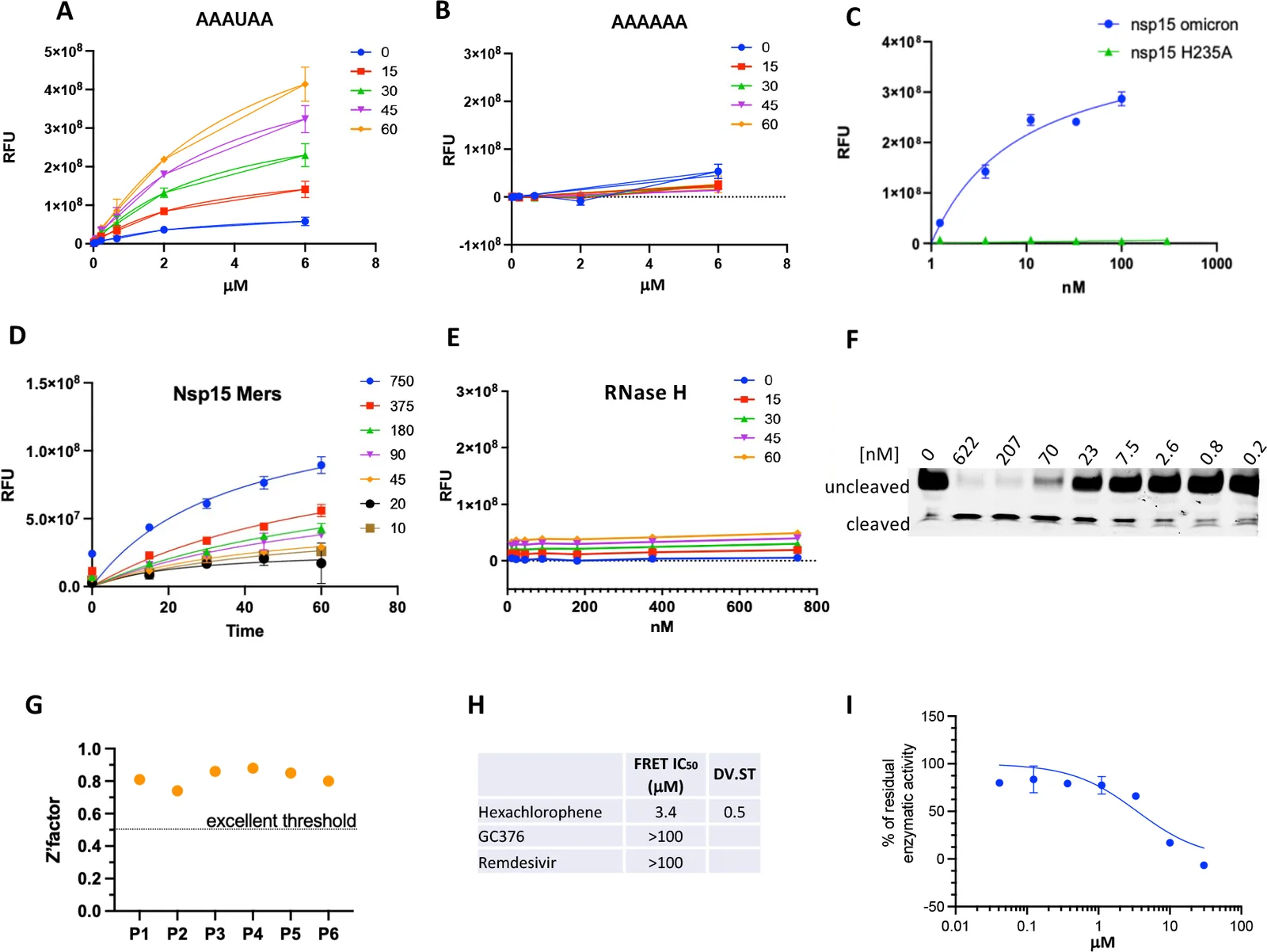
Validation of the FRET-based assay. (**A-B**) Evaluation of Nsp15 ability to cleave different RNA substrates; (**C**) Comparison between active Nsp15 and catalytic mutant H235A; Endonuclease activity of (**D**) MERS-Nsp15 and (**E**) HIV RNase H; (**F**) Nsp15 activity curve in the polyacrylamide gel; (**G**) Z’ factor validation, P=plate; (**H**) FRET assay for Hexachlorophene, GC376, and Rendesivir with IC_50_; (**I**) Hexachlorophene inhibitory curve. Data are expressed as the means ± standard deviations (SDs) from at least three independent experiments.

After optimizing reaction conditions, the signal generated by the reaction of 30 nM Nsp15 and 2 μM substrate increased to ~2 × 10^8^ RFU after 60 min. To quantify kinetic parameters, Michaelis–Menten analysis was performed. The apparent Michaelis constant (K_M_) was calculated as 4.87 μM and the maximum velocity (V_max_) as 7.5 × 10^8^ RFU (Fig. 2A). A non monouridylated ssRNA substrate 5′−6-FAM-AAAAAA-TAMRA-3′ was used as negative control for the reaction (Fig. 2B). We also evaluated the ability of Omicron Nsp15 to cleave different ssRNAs possessing a different ribonucleotide adjacent to the central U (Supplementary Fig. 2A–B). Among the diverse substrates, the sequence, 5′−6-FAM-AAAUAA-TAMRA-3′ yielded the most robust signal, so we decided to use this substrate for HTS.

The presence of the SUMO tag did not affect Omicron Nsp15 activity (Supplementary Fig. 2C). In order to have a strong negative control, site directed mutagenesis of His Omicron Nsp15 was performed to obtain a catalytic dead mutant by introduction of an alanine instead of the histidine in position 235 of the catalytic triad. As expected, the H235A substitution completely abrogated the enzymatic activity (Fig. 2C). MERS Nsp15 was also tested and showed lower enzymatic activity compared to Omicron Nsp15 (Fig. 2D). The endonuclease activity of RNase H associated to the HIV-1 Reverse Transcriptase - that specifically cleaves the RNA strand of an RNA:DNA hybrid duplex - was used as negative control for the reaction, showing no activity in the cleavage of a ssRNA (Fig. 2E).

To confirm Nsp15 enzymatic activity, we performed a complementary polyacrylamide gel-based assay. The assay uses a 14% polyacrylamide gel (19:1 acrylamide:bisacrylamide) supplemented with 7 M urea. An activity curve was generated using increasing concentrations of the Nsp15 Omicron variant, while maintaining the same substrate concentration as used in the FRET assays. As shown in Fig. 2F, the gel reveals a pattern consistent with enzymatic cleavage. The lower band, which increases in intensity with higher enzyme concentrations, corresponds to the cleaved substrate. In contrast, the central band, more prominent in the enzyme-free control, represents the intact (uncleaved) substrate. These results support the enzymatic activity of Nsp15 Omicron and validate the gel-based assay as a useful alternative for monitoring RNA cleavage.

### 3.3. Validation of the FRET assay with Z’ score and known inhibitors

The robustness and reproducibility of the assay were evaluated by calculating the Z′-factor that yielded Z′ values around 0.7 – 0.9, indicating excellent assay quality suitable for high-throughput screening applications (Fig. 2G).

The FRET assay was validated using a known Nsp15 inhibitor as positive control: hexachlorophene (Chen et al., 2023). This compound exhibited IC_50_ values of 3.4 μM (Fig. 2H–I). As negative controls, inhibitors targeting other SARS-CoV-2 proteins, such as GC376 (a protease inhibitor) and remdesivir (a polymerase inhibitor), were tested. As expected, these compounds showed no inhibitory activity (Fig. 2H).

### 3.4. Implementation of FRET assay protocol in 1536-well microplate format

**Figure 3.**
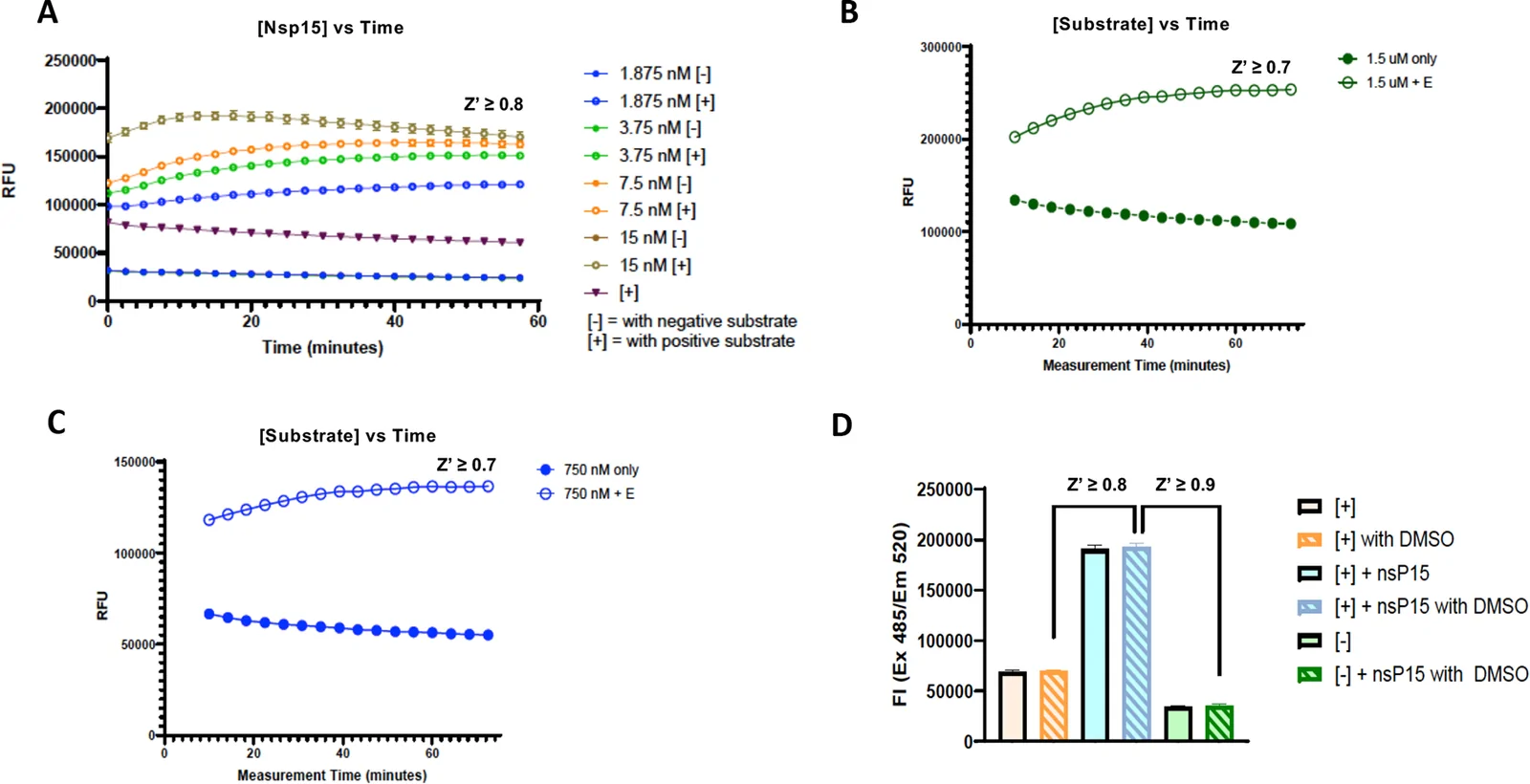
Determination of the performance of the FRET-based Nsp15 assay in a high-throughput format. **(A)** Reproduction of FRET assay in 1536-wpf and Z’-factor calculation; **(B-C)** Substrate vs time curves and Z’-factor calculation; **(D)** DMSO tolerance assay. The graphs (A-C) were plotted with average of 128 wells/condition and SD was shown as error bar. Bar graph D was created with average of 4–664 wells/condition and SD was shown as error bar.

The FRET assay was then implemented to a 1536-well plate format, with time-course experiments conducted across a range of enzyme and substrate concentrations. To adapt the assay to the 1536-wpf, we evaluated Nsp15 EndoU activity at concentrations between 1.875 and 15 nM (Fig. 3A). Well-defined reaction curves were produced. Low concentrations of Nsp15 were sufficient to generate clear reaction curves, with the reaction reaching a plateau at approximately 20 min. Based on these findings, we used 7.5 nM Nsp15 for future screening. Statistical analysis was performed confirming assay robustness and accuracy. To minimize costly substrate consumption, we selected a substrate concentration of 0.75 μM for HTS, as it provided excellent assay performance (Fig. 3B and C). Z’ score experiment was performed to confirm the robust statistical parameters (Fig. 3C).

To identify an appropriate range of DMSO concentrations to be used for our screening purpose, we performed a DMSO tolerance experiment. The assay demonstrated tolerance to 0.56% final DMSO concentration, with no impact on performance (Fig. 3D). Importantly, the assay showed no evidence of false positives, further confirming its reliability for HTS applications (Supplementary Fig. 3).

### 3.5. HTS LOPAC screening

**Figure 4.**
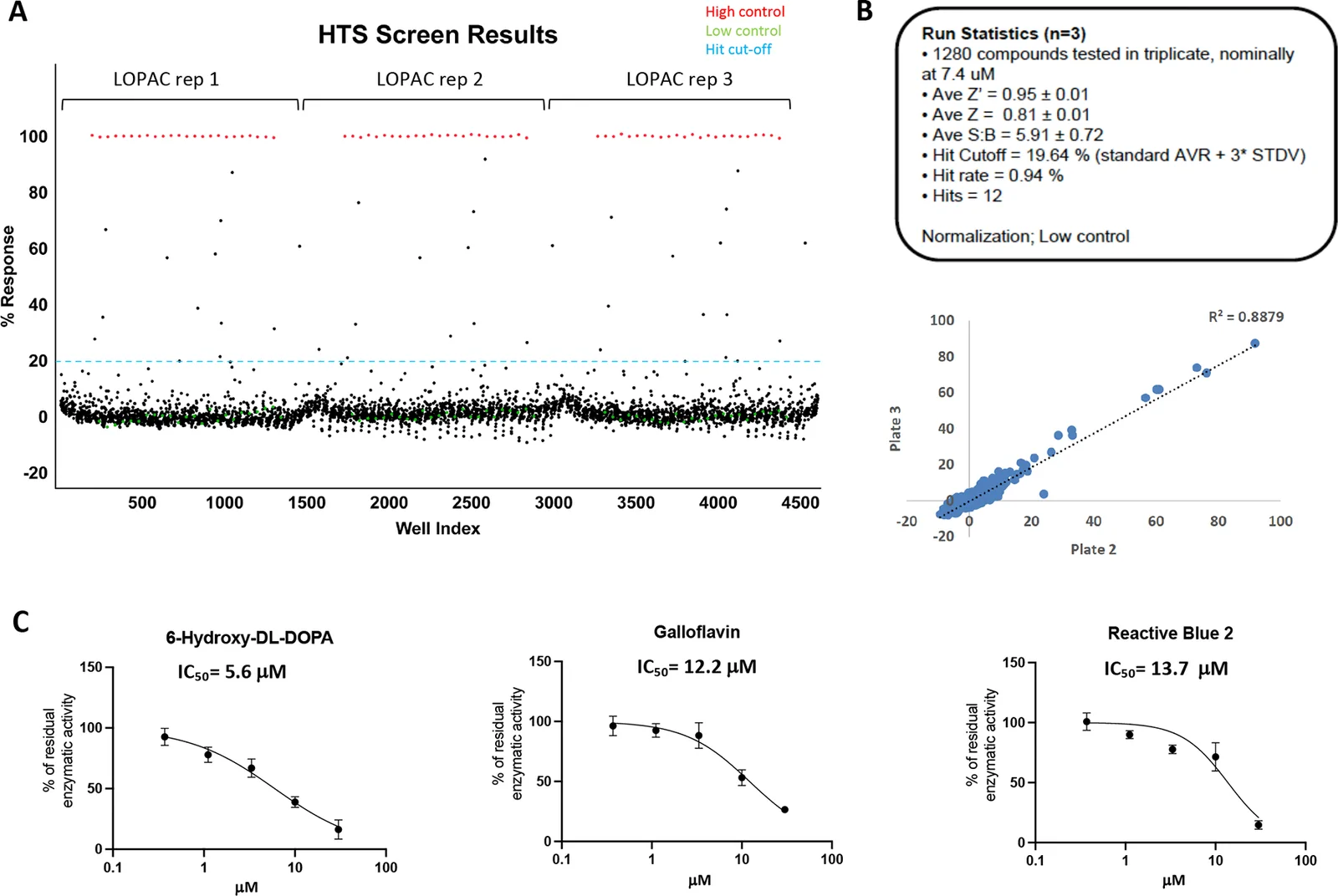
High-throughput screen results. **(A)** LOPAC screening, three replicates (rep 1, rep2, rep3); **(B)** *top:* Statistics for LOPAC screening, *bottom:* Correlation plot was performed to compare percentage (%) response of 1280 compounds between two plates and R^2^ was calculated; **(C)** Curves with IC_50_ of most active compounds. Data are expressed as percentage (%) of residual enzymatic activity and represent the means ± standard deviations (SDs) from three independent experiments.

To test the feasibility of our approach, a LOPAC^®^ (Library of Pharmacologically Active Compounds) pilot run of 1280 compounds was tested in triplicate, at a concentration of ~7.4 μM (Fig. 4A), in the 1536-well plate format. The assay exhibited excellent Z’ scores across three replicate plates (Fig. 4B). Based on a mathematical algorithm to determine active compounds (the average + 3x *stdev* of all samples tested not including outliers that are 3x *stdev* above the high controls or 3x *stdev* below the low controls), the hit rate was determined to be 0.94%. The result of all three assay plates correlated very well, which is indicated as example plot with R^2^ = 0.89 at the bottom of Fig. 4B. We selected 12 hit compounds based on a hit cutoff of 19.64% activity (Supplementary Fig. 4A–B), and we ran the dose-response curved in the 384-assay format. Among them, 6-Hydroxy-DL-Dopa, Galloflavin, and Reactive Blue 2, showed IC_50_ values ranging between 5.6 and 13.7 μM (Fig. 4C), and were considered for further cellular characterization.

### 3.6. Evaluation of antiviral activity of Nsp15 inhibitors in cellular models

**Figure 5.**
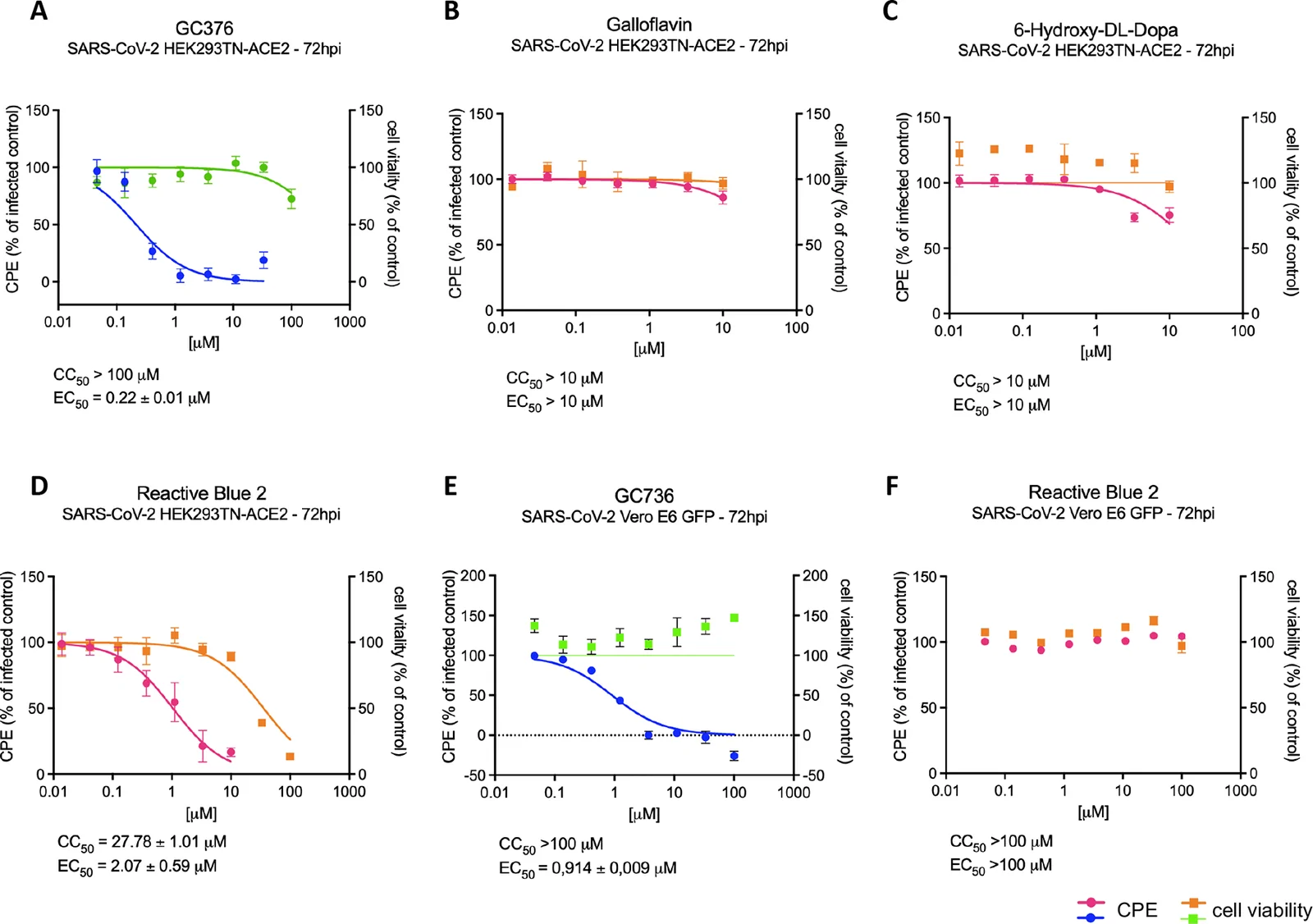
Antiviral activity of SARS-CoV-2 Nsp15 inhibitors. SARS-CoV-2 replication assay in HEK293TN-ACE2 for **(A)** GC736, **(B)** Galloflavin, **(C)** 6-Hydroxy-DL-Dopa, **(D)** Reactive Blue 2; Replication assay in Vero E6 GFP for **(E)** GC736, **(F)** Reactive Blue 2. Plots show the % of cytopathic effect (CPE) and the % of cell viability after infection with SARS-CoV-2 and treatment with increasing concentrations of compounds. Data are expressed as the mean ± SD of at least two independent experiments in triplicate. CC_50_ and EC_50_ values are indicated above the curves and were calculated using Graphpad.

The selected compounds were tested in two cellular systems to assess their ability to inhibit SARS-CoV-2 replication, using GC376 as a positive control for viral inhibition (Fig. 5A).

The first biological model employed HEK293TN-ACE2 cells. These cells possess a fully functional innate immune response, making them a suitable model to investigate both roles of Nsp15: (i) its endoribonuclease activity, which facilitates viral replication, and (ii) its immunomodulatory function, which suppresses host antiviral responses. Among the 3 compounds tested, Reactive Blue was confirmed to inhibit SARS-CoV-2 replication in this model, with an EC_50_ of 2 μM (Fig. 5B–D), with a selective index (SI) of 13, while the other two compounds were inactive in this viral replication model.

Reactive Blue 2 was subsequently evaluated in a second cellular system based on Vero E6 cells. This model is particularly informative because Vero E6 cells lack type I IFN production, resulting in a defective innate immune response (Naoki et al., 2014). As a result, the cellular defense pathways typically blocked by Nsp15 endoribonuclease activity are already inactive in these cells. Thus, this system allows for selective evaluation of Nsp15 role in innate immune evasion. If the compound’s antiviral effect relies on restoring host antiviral signaling suppressed by Nsp15, then the absence of such activity in this IFN-deficient model would support that mechanism of action. Consistent with this hypothesis, the compound showed no antiviral activity in Vero E6 cells (Fig. 5F), confirming the correlation between antiviral activity and innate immune response restoration. As positive control for inhibition GC736 was used, being able to inhibit SARS-CoV-2 also in Vero E6 (Fig. 5E). To exclude a difference in permeability of Reactive Blue 2 between the two cellular models, Reactive Blue 2 content was analyzed by HPLC in cell lysates. As shown by the HPLC-DAD chomatograms, the pick corresponding to Reactive Blue 2, observable at 2.8 min at 620 nm as for reference control (Reactive Blue 2 alone) (Supplementary Fig. 5A–B), was detected either in HEK (Supplementary Fig. 6A–B) and Vero lysates (Supplementary Fig. 7A–B), confirming no difference in absorption of the compound in the two different cell lines. To confirm this finding, we measured absorbance at 595 nm in the extracellular medium at Time 0 (T0) and T24 h post-treatment, assessing a similar decrease of Reactive Blue 2 in the two cell lines (Supplementary Fig. 8).

### 3.7. Assessment of Nsp15 and Reactive Blue 2 binding

**Figure 6.**
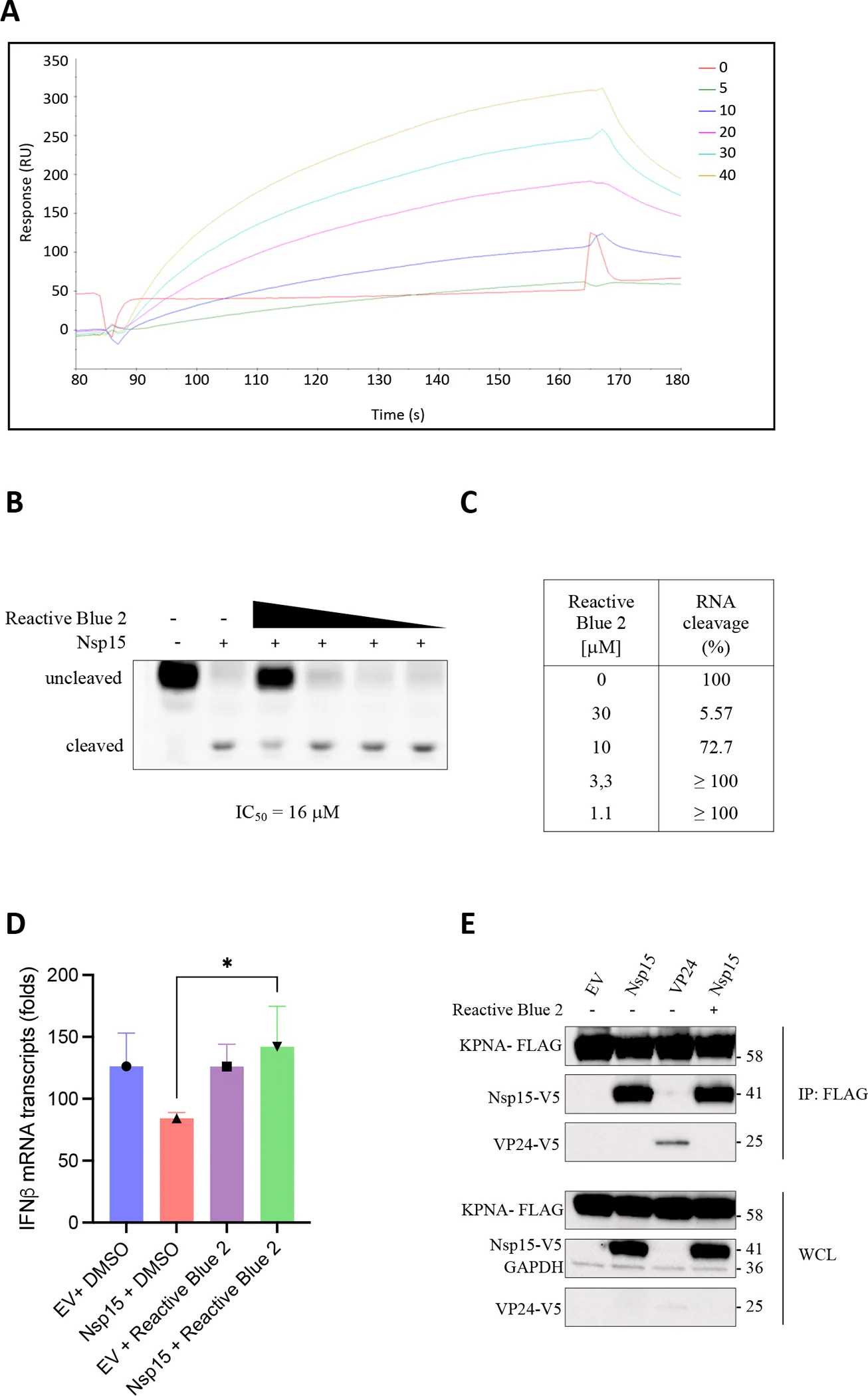
Reactive Blue 2 inhibits Nsp15 restoring IFN production. **(A)** Assessment of binding between Nsp15 and Reactive Blue 2 by surface-plasmon resonance. Nsp15 and Reactive Blue 2 interaction was captured on the sensor chip and different concentrations of reactive Blue 2 are expressed with different colors in legend (μM); **(B)** Reactive Blue 2 inhibits RNA cleavage mediated by Nsp15 in the gel-based assay; **(C)** percentage of RNA cleavage in presence of Nsp15 alone and decreasing concentrations of Reactive Blue 2; **(D)** Inhibition of IFNβ transcription by Nsp15 after SeV stimulation in HEK293T cells; mRNA expression levels are expressed as folds of mRNA transcripts in stimulated samples relative to not stimulated controls, *, P =0.039, two-tailed unpaired Student’s t test (n=3); **(E)** Co-IP of Nsp15-V5 and KPNA-FLAG in presence or not of Reactive Blue 2; Ebola VP24-V5 was used as positive control for immunoprecipitation with KPNA-FLAG.

To confirm the binding between Nsp15 and Reactive Blue 2, we performed an SPR-based ligand binding assay. As shown in Fig. 6A, we confirmed binding of Nsp15 with the compound. We calculated the K_D_ that showed a value of 13.78 μM, that was perfectly aligned with the IC_50_ value we obtained in the FRET assay. The binding was also confirmed using microscale thermophoresis (MST) (Supplementary Fig. 9A). In contrast, no evidence of binding was observed between Reactive Blue 2 and the RNA substrate (Supplementary Fig. 9B).

### 3.8. Restoration of IFN response mediated by Reactive Blue 2

It is known that Nsp15 mediates evasion of IFN response by processing viral dsRNA, a genome replication intermediate, via its uracil-specific endoribonuclease activity, and preventing recognition by cellular dsRNA sensors(Otter et al., 2024). This finally leads to the inhibition of the IFN expression. We then wanted to confirm the role of EndoU Nsp15 in blocking the IFN system in cells and assess the effect of Reactive Blue 2 on the cascade. Since Reactive Blue 2 mechanism of action was the inhibition of Nsp15-mediated cleavage of RNA, which was confirmed also with the urea gel-based assay (Fig. 6B–C), we hypothesized that the compound was consequently able to restore the IFN expression, abrogated by EndoU Nsp15. To confirm this hypothesis, HEK293T cells were transfected with Nsp15 or Empty Vector (EV). The following day, the IFNβ production was stimulated by infection with SeV, which is known to activate a strong IFN response, and cells were treated with DMSO or with the compound (10 μM). The IFN-β transcripts were quantified by RT-qPCR. This assay serves as an indirect method to assess Nsp15 catalytic activity in cells and moreover to determine whether Reactive Blue 2, being able to block the Nsp15 can counteract this inhibition, thus restoring the mRNA IFNβ levels.

Our results showed that Nsp15 was able to inhibit the IFNβ expression activated by SeV (Fig. 6D). We found that the treatment of cells with Reactive Blue 2 resulted in a significant restoration of the IFNβ expression (around 80%). Beyond the EndoU function, Nsp15 also interacts with KPNA1, that is implicated in the nuclear transport of transcription factors driving the IFNβ expression. To exclude that Reactive Blue 2 was acting blocking this interaction, we performed a co-immunoprecipitation of Nsp15 and KPNA1 in presence of compound, using Ebola VP24 as positive control for co-immunoprecipitation with KPNA1(Fanunza et al., 2020). As expected, we observed that Reactive Blue 2 was not interfering at this step of the cascade, confirming its specificity for the inhibition of the Endo U activity. In conclusion, our findings confirmed the role of the compound in blocking SARS-CoV2 replication by specifically targeting Nsp15 endonuclease domain and restoring the IFNβ cascade.

## 4. Discussion

The growing understanding of innate immune mechanisms has led to the current challenge of restoring the innate pathways, a promising therapeutic strategy for combating viral infections (Fanunza and Corona, 2023).

To the best of our knowledge the known inhibitors of Nsp15 have been tested on their ability to block the endonuclease activity, but none have been tested on their ability to restore the IFN response in cells (Van Loy et al., 2024).

The first compounds reported to inhibit SARS-CoV-2 Endo U Nsp15 were Tipiracil and Exebryl-1 (Choi et al., 2021; Kim et al., 2021). However, they were weak inhibitors of the viral replication with EC_50_ values of 50 and 66 μM, respectively. Following them, hexachlorophene, IPA-3, and a few hits from the ChemBridge DIVERSet library were also showed to inhibit the EndoU activity in the low micromolar range(Chen et al., 2023). Among them, only hexachlorophene showed to be a potent SARS-CoV-2 inhibitor with an EC_50_ value around 1 μM and CC_50_ value of 15 μM. More recently a few other studies reported the ability of small molecules to block the EndoU activity, however with none or low micromolar potency in the antiviral replication assay (Bajaj et al., 2025; Chatziefthymiou et al., 2025; Mehyar et al., 2025; Rampias et al., 2024).

We aimed to identify compounds capable of SARS-CoV-2 Nsp15 dual inhibitory activity: selective inhibition of endonuclease activity of Nsp15, thereby blocking viral replication, and simultaneously impairing the protein’s role in evading the host innate immune response. Firstly, by carefully optimizing enzyme/substrate concentrations, cofactor selection, buffer conditions, and reaction components, we optimized a reliable and sensitive FRET-based assay. This platform was fully validated and implemented to a HTS format. Hence, we used the commercially available LOPAC library for screening to identify potential inhibitors against the Endo U catalytic activity of Nsp15. Setting a hit cut-off of 19%, we identified from 12 hits with inhibitory properties against the viral endoribonuclease. Among them, Reactive blue 2 was the most promising molecule, blocking SARS-CoV-2 replication with a low EC_50_ = 2 μM and low cytotoxicity (CC_50_ > 28 μM).

Reactive Blue 2 is an anthraquinone derivative with a molecular weight of 774.16 g/mol. It is primarily known as a P2 receptors antagonist with IC_50_ values ranging from 7.7 to 35.5 μM based on the Reactive Blue 2 mixture analyzed (Glänzel et al., 2003; Tuluc et al., 1998). It is found to be a potent inhibitor of a protein kinase isolated and purified from thylakoids (IC_50_ 5 μM) (Coughlan et al., 1991). With a predicted Log P between 1.36 and 7.10, it is generally considered a poor membrane permeant, even if there are studies confirming the Reactive Blue 2 ability to cross the cell membranes and inhibit intracellular enzymes (Claes et al., 2004; Kaneko et al., 2023). Cytotoxicity investigations *in vitro* demonstrated no toxicity for Reactive Blue 2. Genotoxic effects were not observed in isolated human skin fibroblasts and dermal 3D models (Leme et al., 2014; Zini Moreira Silva et al., 2022).

Nsp15 is dispensable for efficient virus replication in an immune-deficient environment. It has been recently reported that SARS-CoV-2 Nsp15 EndoU mutants’ replication is severely attenuated in immune-competent cells, while they replicate as efficiently as WT virus in Vero E6 Cells, which lack an intact IFN system (Caobi et al., 2025). In line with these observations, we demonstrated that Reactive Blue 2 inhibits viral replication in an IFN context dependent manner. In fact, we confirmed the compound to be a selective inhibitor of SARS-CoV-2 in HEK293TN-ACE2 cells. Notably, this inhibitory effect was absent in Vero E6 cells, confirming that the antiviral action of Reactive Blue 2 was dependent on a functional IFN response. Consistent with this result, Reactive Blue 2 was also able to restore the Nsp15 IFN response inhibition, specifically targeting the EndoU catalytic step. It is worth noting that Reactive Blue 2 has been also reported to be able to inhibit the methyltransferase activity of SARS-CoV-2 Nsp14 (Kasprzyk et al., 2021). Nsp14 has a known involvement in the innate immune evasion (Yuen et al., 2020). Hence, Reactive Blue 2, on the one side, may have two different viral targets both involved in the viral immune evasion, on the other side, by restoring the type I IFN response, the compound triggers a cascade that restricts viral replication inducing an antiviral state. This suggested positive cooperativity may explain the lower EC_50_ value (2 μM) observed in the antiviral assay as compared to the IC_50_ value (13.7 μM) measured in the biochemical assay.

Overall, this study provides proof of concept for the rational design of biologically active compounds targeting Nsp15 and its role of IFN antagonism. Our results strongly support that the inhibition of viral replication is directly linked to Nsp15 and the restoration of innate immunity. Importantly, this is the first inhibitor demonstrated to block SARS-CoV-2 via this mechanism of action. Taking all this into consideration, we provided novel insight on the emergence of the endoribonuclease as a promising drug target for future COVID-19 treatment.

## Supplementary Material

Supplementary data Maloccu et al., 2026

Supplementary table, Maloccu et al 2026

## Data Availability

Data will be made available on request.

## Appendix A. Supplementary data

Supplementary data to this article can be found online at https://doi.org/10.1016/j.antiviral.2026.106443.
