# Supplementary material for: Identification of Nsp15 inhibitors restoring interferon-dependent antiviral activity against SARS-CoV-2
Source: Antiviral Res. Author manuscript; Available in PMC 2026 Sep 8. (PMC13551574; doi:10.1016/j.antiviral.2026.106443)
Supplement: Supplementary file 1 [file NIHMS2206332-supplement-Supplementary_data_Maloccu_et_al___2026.pdf]

Identification of Nsp15 Inhibitors Restoring Interferon-Dependent Antiviral Activity Against SARS-CoV-2

Stefania Maloccu[1], Yuka Otsuka[2], Elizabeth Molchan[2], Michelle Foti[1], Annalaura Paulis[1], Jyoti Vishwakarma[3], Paolo Malune[1], Filippo Cottiglia[1], Angela Corona[1], Louis Scampavia[2], Timothy P. Spicer[2], Reuben S. Harris[3,4], Enzo Tramontano[1#*], Elisa Fanunza[1#*]

[1]Department of Life and Environmental Science, University of Cagliari, Cagliari, Italy [2] Department of Molecular Medicine, The Herbert Wertheim UF Scripps Institute for Biomedical Innovation & Technology, Jupiter, Florida, USA, [3] Department of Biochemistry and Structural Biology, University of Texas Health San Antonio, San Antonio, Texas, USA, [4] Howard Hughes Medical Institute, University of Texas Health San Antonio, San Antonio, Texas, USA.

# These authors share last authorship

* Corresponding authors: tramon@unica.it, elisa.fanunza@unica.it

Supplementary Data

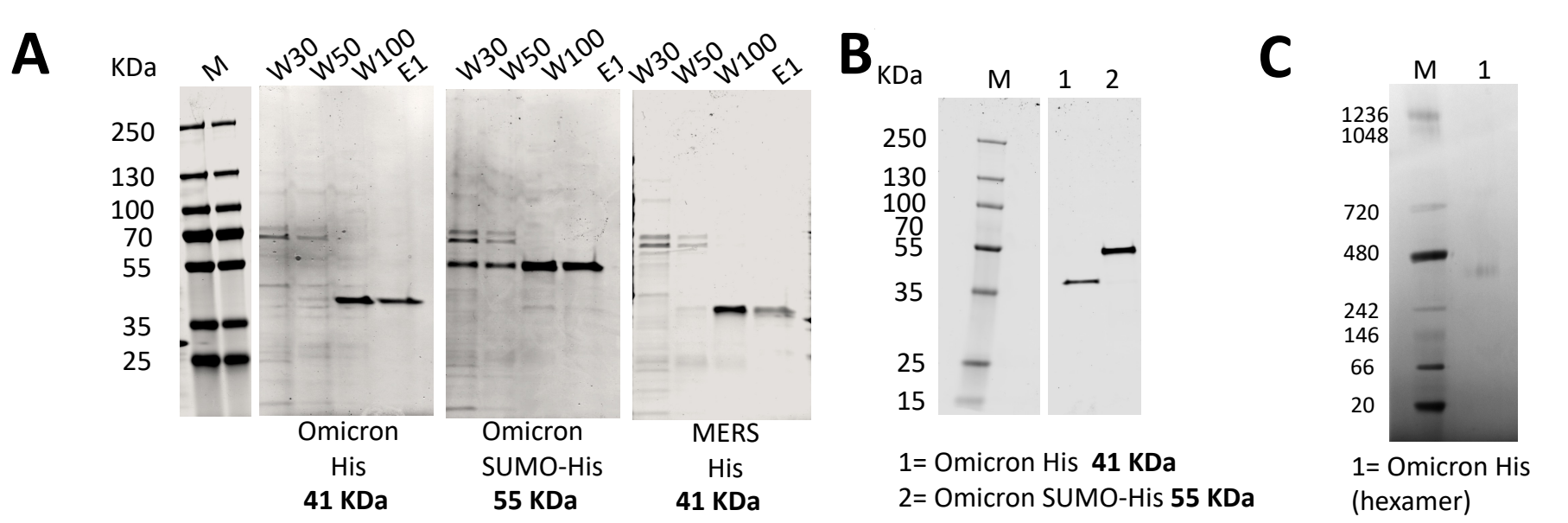

Supplementary Figure 1. SDS-Page and Western Blot of recombinant Nsp15 (**A**) SDS-PAGE gel with purified Nsp15: M, marker; W30-50-100, Wash 30-50-100 mM Imidazole, E1, Elution 300 mM Imidazole; (**B**) Western Blot of Omicron Nsp15; (**C**) Native page with purified hexameric Nsp15.

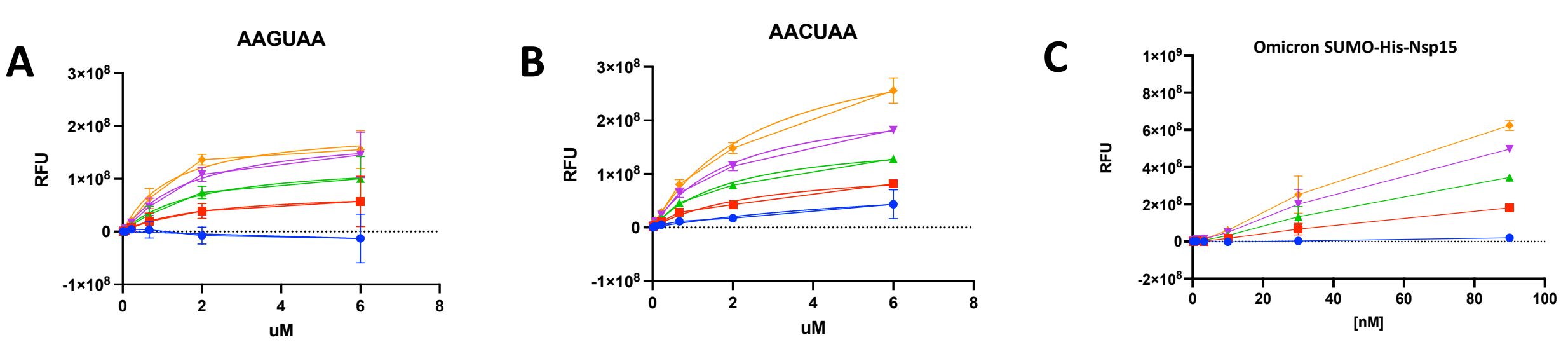

Supplementary Figure 2. Curves of Nsp15 endonuclease activity using the FRET-based assay. (**A-B**) Evaluation of Nsp15 ability to cleave different RNA substrates; EndoU activity of (**C**) SUMO-His-Nsp15

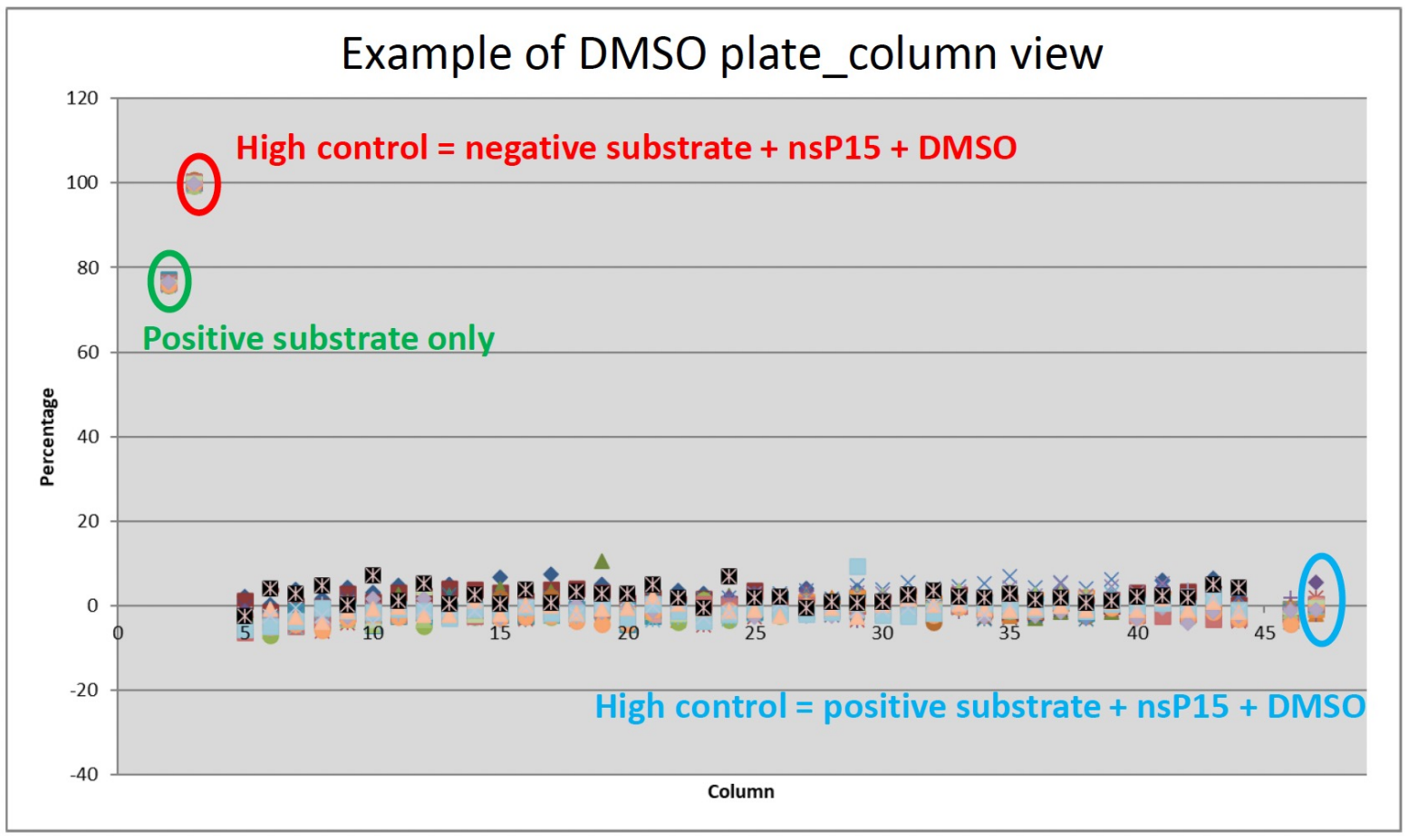

%CV	S/B	Z	Z'
1.81	5.96	0.922	0.939

3*SD	Avg + 3*SD	Hit Cutoff	Hits
6.6	6.1	20.0	0

Supplementary Figure 3. Evaluation of presence of false negatives in the FRET-based screening assay.

A

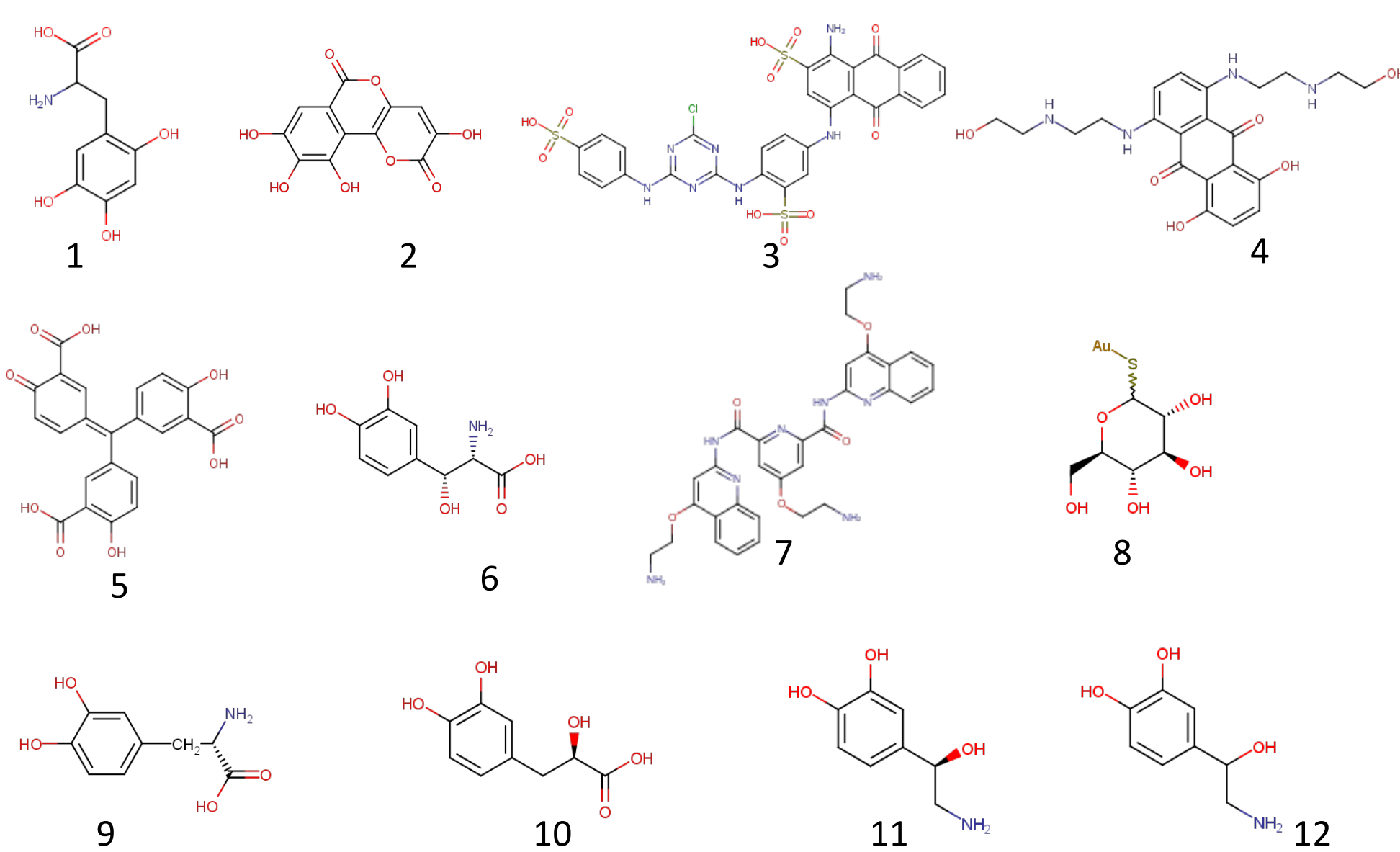

B

	Name compound
1	6-Hydroxy-DL-DOPA
2	Galloflavin
3	Reactive Blue 2
4	Mitoxantrone dihydrochloride
5	Aurintricarboxylic acid
6	L-DOPS
7	Pyridostatin hydrochloride
8	Aurothioglucose hydrate
9	3,4-Dihydroxy-L-phenylalanine
10	Danshensu sodium salt
11	L-(−)-noradrenalina
12	(±)-Norepinephrine (+)-bitartrate salt

Supplementary Figure 4. Active compounds from HTS LOPAC screening (**A**) LOPAC hits structures and **(B)** names.

A

Qualitative - Detailed Report

Agilent

**Sequence Name:**	SingleSample		
**Data file:**	Procyon blue ref.dx	**Project Name:**	HPLC_DAD
**Sample name:**	Procyon blue ref	**Operator:**	SYSTEM (SYSTEM)
**Instrument:**	HPLC_DAD	**Acquired on:**	2026-04-17 20:04:03+02:00
**Inj. volume:**	6.000 µL	**Location:**	1
**Acq. method:**	blu procione.amx		
**Processing method:**	*3D UV Quantitative_DefaultMethod.pmx		

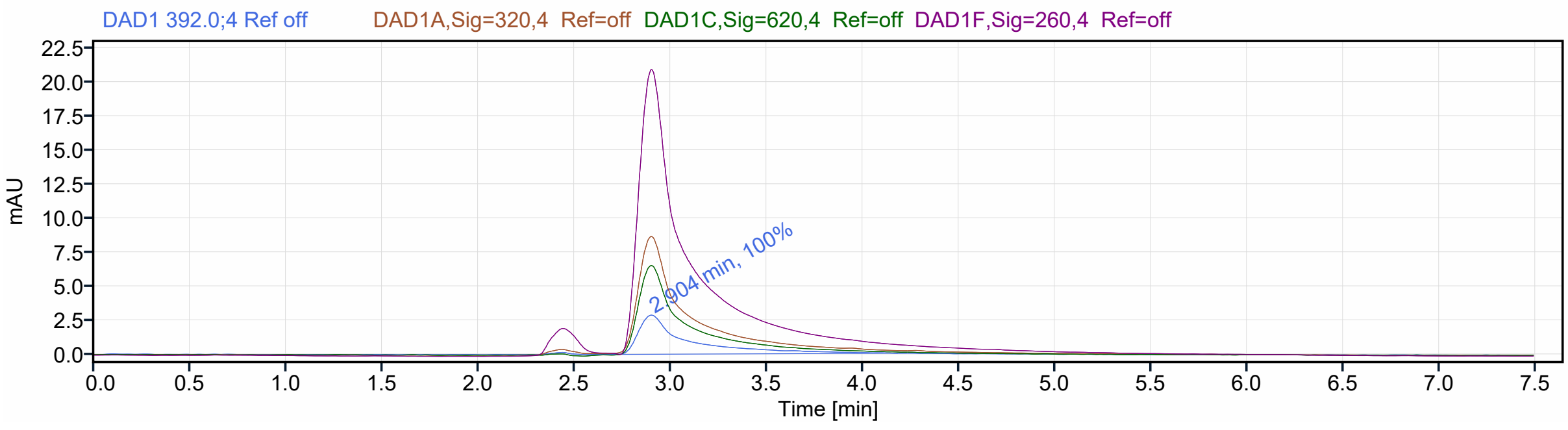

Peak Results (Area Percent at least 1%)

RT (min)	Signal Description	Width (min)	Area	Height	Area%
2.444	DAD1F,Sig=260,4 Ref=off	0.578	21.3	2.0	4.74
2.904	DAD1A,Sig=320,4 Ref=off	1.546	146.9	8.6	100.00
2.904	DAD1C,Sig=620,4 Ref=off	2.268	126.7	6.6	100.00
2.904	DAD1 392.0;4 Ref off	1.492	51.1	2.9	100.00
2.905	DAD1F,Sig=260,4 Ref=off	3.335	428.2	21.1	95.26

**Sum DAD1 392.0;4 Ref off**	51.1
**Sum DAD1A**	146.9
**Sum DAD1C**	126.7
**Sum DAD1F**	449.5

B

Peak Details (Area Percent at least 1%)

Signal Description: DAD1A,Sig=320,4 Ref=off

Spectra overlay at peak apex, up and down slopes

Peak RT: 2.904 min **Area %:** 100.00%

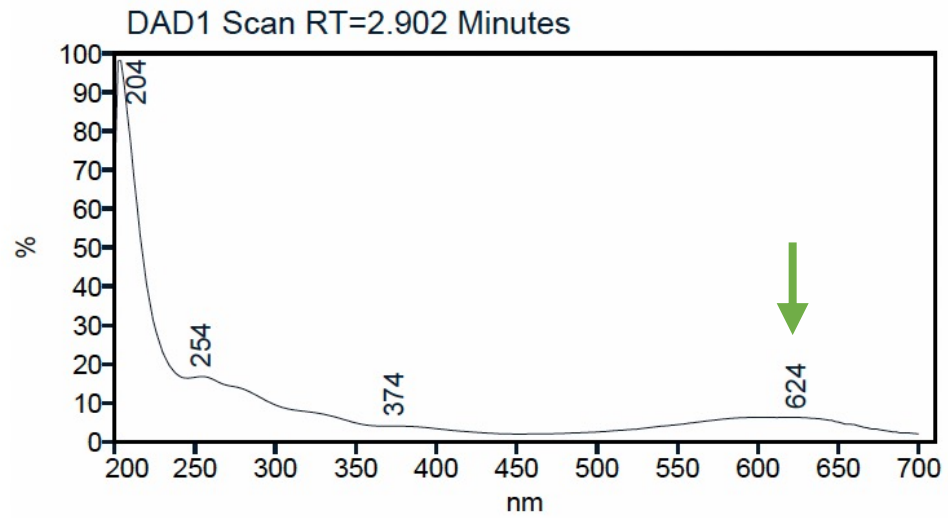

Supplementary Figure 5. (A) HPLC-DAD chromatogram for compound Reactive Blue 2, (**B**) Peak detail with green arrow indicating pick of Reactive Blue 2 at 620 nm.

A

Qualitative - Detailed Report

Agilent

**Sequence Name:**	SingleSample		
**Data file:**	HEK cells	**Project Name:**	HPLC_DAD
**Sample name:**	HEK cells	**Operator:**	SYSTEM (SYSTEM)
**Instrument:**	HPLC_DAD	**Acquired on:**	2026-04-17 19:41:20+02:00
**Inj. volume:**	30.000 µL	**Location:**	1
**Acq. method:**	blu procione.amx		
**Processing method:**	*3D UV Quantitative_DefaultMethod.pmx		

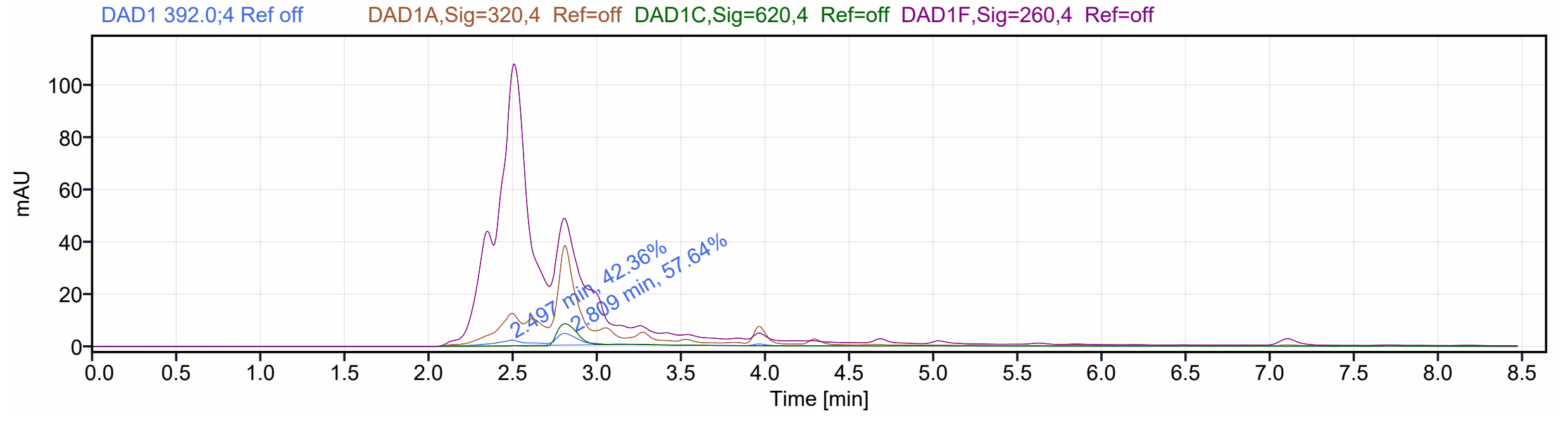

Peak Results (Area Percent at least 1%)

RT (min)	Signal Description	Width (min)	Area	Height	Area%
2.350	DAD1F,Sig=260,4 Ref=off	0.320	272.0	41.8	14.54
2.497	DAD1A,Sig=320,4 Ref=off	0.503	113.6	11.4	21.62
2.497	DAD1 392.0;4 Ref off	0.639	29.3	2.1	42.36
2.509	DAD1F,Sig=260,4 Ref=off	0.330	1098.3	104.6	58.70
2.617	DAD1A,Sig=320,4 Ref=off	0.145	65.2	9.0	12.40
2.808	DAD1F,Sig=260,4 Ref=off	0.400	468.4	43.3	25.03
2.809	DAD1 392.0;4 Ref off	0.458	39.9	4.4	57.64
2.811	DAD1A,Sig=320,4 Ref=off	0.281	264.4	36.5	50.31
2.811	DAD1C,Sig=620,4 Ref=off	0.457	75.3	8.5	100.00
3.053	DAD1A,Sig=320,4 Ref=off	0.177	26.4	4.2	5.03
3.272	DAD1A,Sig=320,4 Ref=off	0.207	12.9	2.7	2.46
3.965	DAD1A,Sig=320,4 Ref=off	0.259	32.9	6.6	6.26
4.293	DAD1A,Sig=320,4 Ref=off	0.283	10.2	2.1	1.93
7.106	DAD1F,Sig=260,4 Ref=off	0.653	19.1	2.5	1.02

**Sum DAD1 392.0;4 Ref off**	69.3
**Sum DAD1A**	525.6
**Sum DAD1C**	75.3
**Sum DAD1F**	1857.8

Supplementary Figure 6A. HPLC-DAD chromatogram for HEK293T lysates

B

Qualitative - Detailed Report

Peak Details (Area Percent at least 1%)

Signal Description: DAD1A,Sig=320,4 Ref=off

Spectra overlay at peak apex, up and down slopes

Peak RT: 2.497 min **Area %:** 21.62%

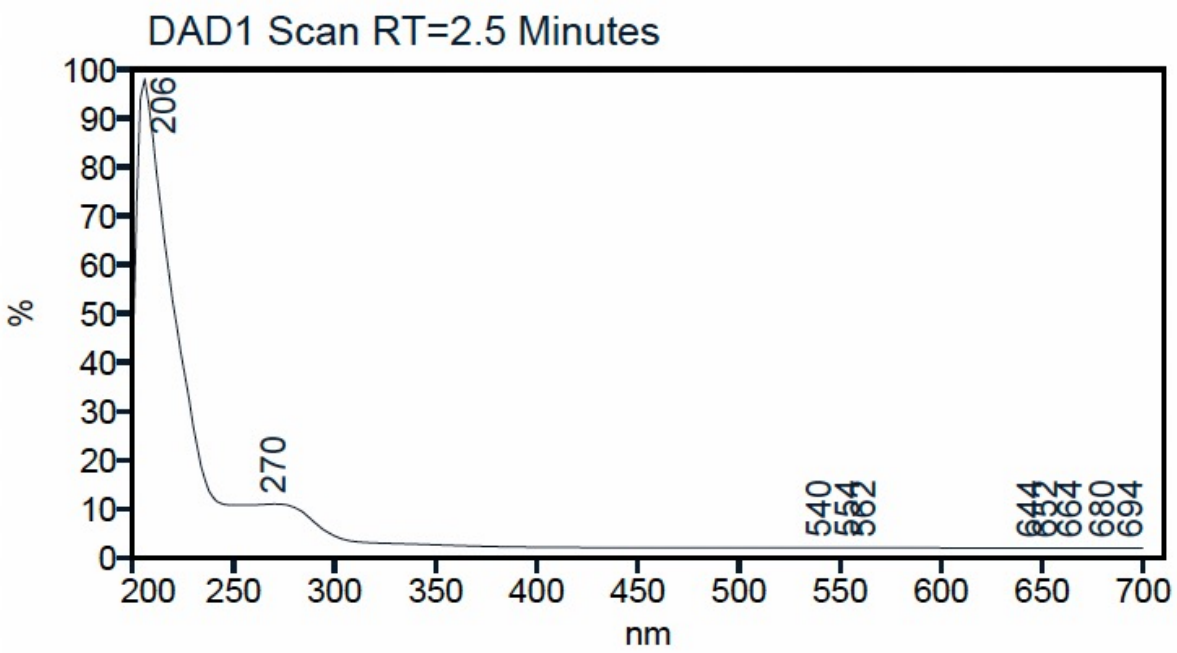

Peak RT: 2.617 min **Area %:** 12.40%

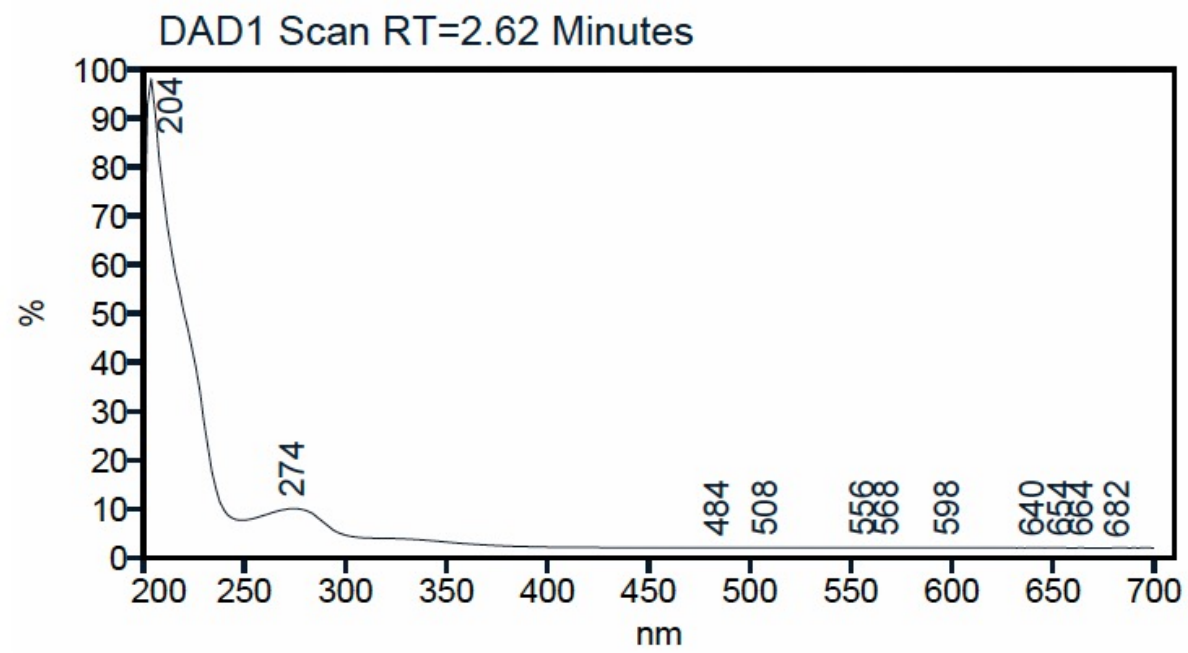

Peak RT: 2.811 min **Area %:** 50.31%

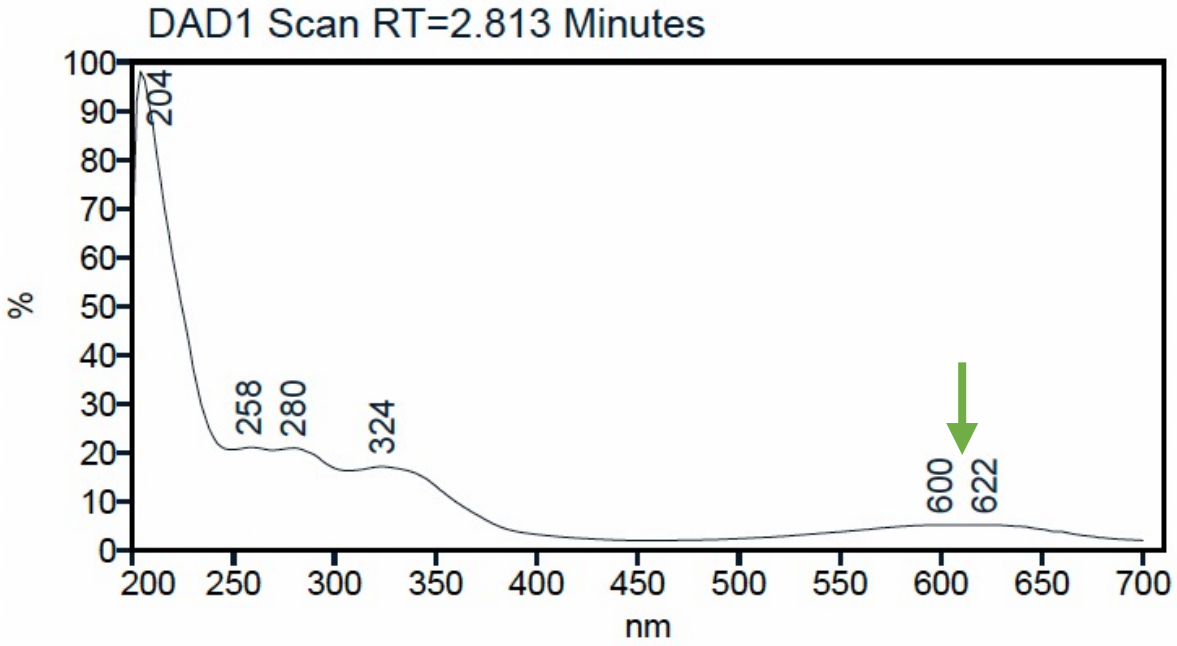

Peak RT: 3.053 min **Area %:** 5.03%

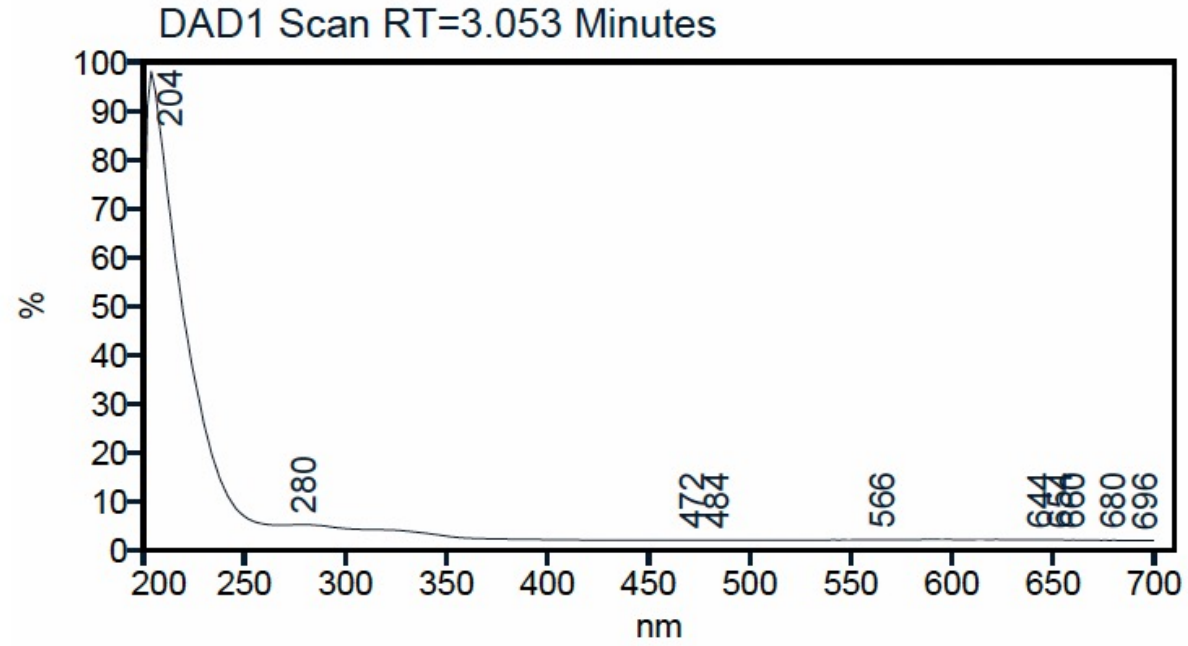

Peak RT: 3.272 min **Area %:** 2.46%

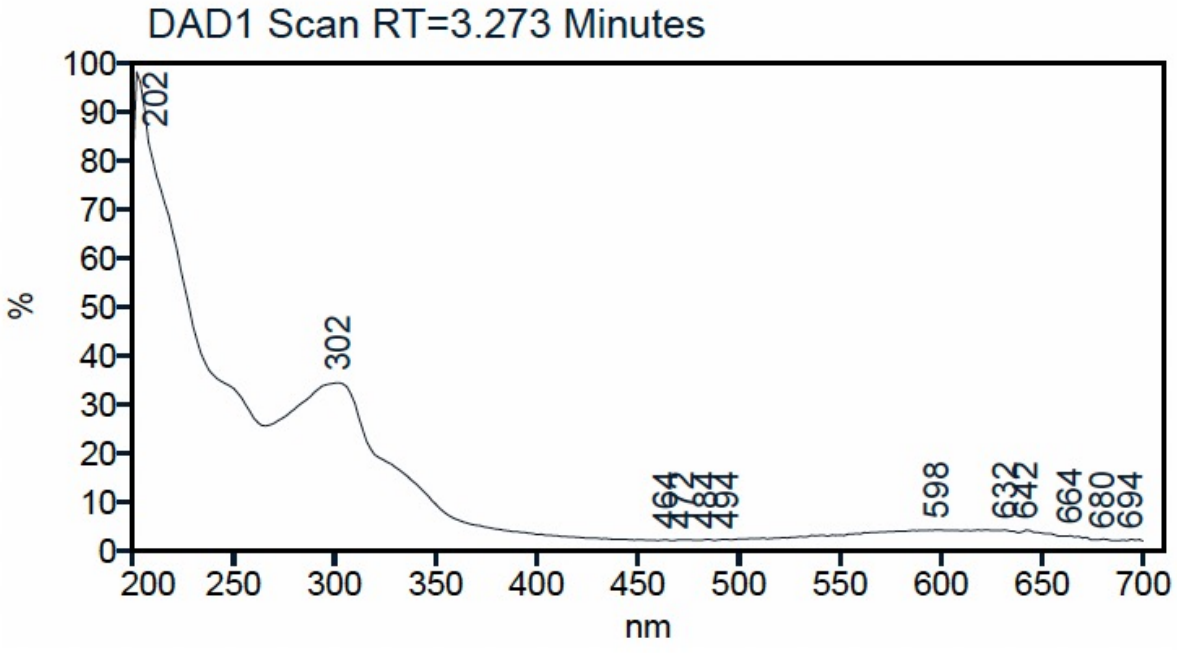

Peak RT: 3.965 min **Area %:** 6.26%

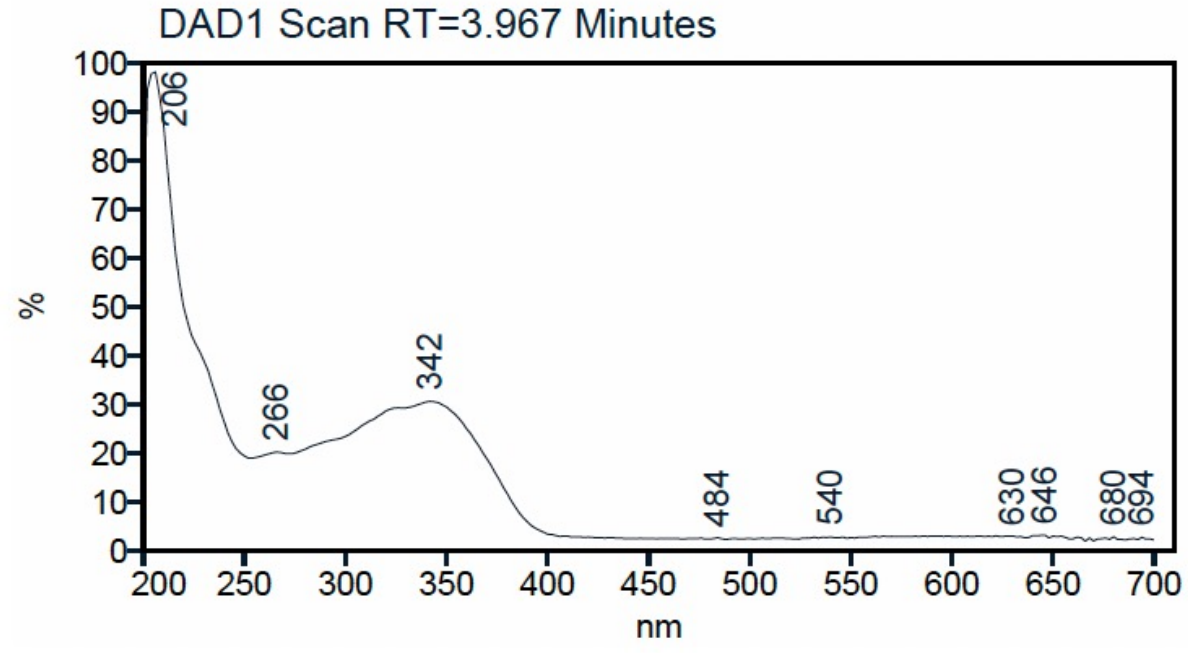

Supplementary Figure 6B. Peak details for HPLC-DAD chromatogram for HEK293T lysates, green arrow indicates pick of Reactive Blue 2.

A

Qualitative - Detailed Report

Agilent

**Sequence Name:**	SingleSample		
**Data file:**	Vero cells	**Project Name:**	HPLC_DAD
**Sample name:**	Vero cells	**Operator:**	SYSTEM (SYSTEM)
**Instrument:**	HPLC_DAD	**Acquired on:**	2026-04-17 18:14:14+02:00
**Inj. volume:**	30.000 µL	**Location:**	1
**Acq. method:**	blu procione.amx		
**Processing method:**	*3D UV Quantitative_DefaultMethod.pmx		

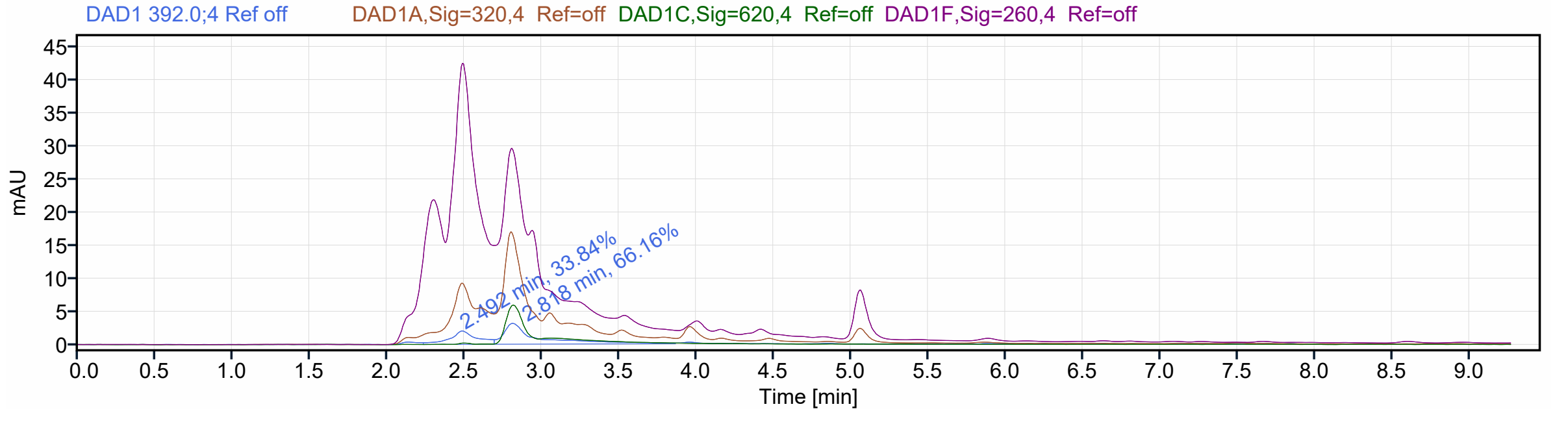

Peak Results (Area Percent at least 1%)

RT (min)	Signal Description	Width (min)	Area	Height	Area%
2.307	DAD1F,Sig=260,4 Ref=off	0.350	203.4	21.1	18.33
2.491	DAD1A,Sig=320,4 Ref=off	0.386	56.3	7.3	27.50
2.492	DAD1 392.0;4 Ref off	0.459	24.7	2.0	33.84
2.496	DAD1F,Sig=260,4 Ref=off	0.313	460.6	41.2	41.51
2.611	DAD1A,Sig=320,4 Ref=off	0.116	19.1	3.2	9.33
2.808	DAD1A,Sig=320,4 Ref=off	0.309	106.1	14.1	51.82
2.812	DAD1F,Sig=260,4 Ref=off	0.221	259.4	27.5	23.38
2.818	DAD1 392.0;4 Ref off	1.172	48.3	3.1	66.16
2.822	DAD1C,Sig=620,4 Ref=off	0.297	45.9	5.9	100.00
2.944	DAD1F,Sig=260,4 Ref=off	0.556	142.1	14.7	12.80
3.964	DAD1A,Sig=320,4 Ref=off	0.234	10.6	1.9	5.17
5.064	DAD1F,Sig=260,4 Ref=off	0.387	44.2	7.4	3.98
5.064	DAD1A,Sig=320,4 Ref=off	0.410	12.7	2.1	6.18

**Sum DAD1 392.0;4 Ref off**	73.0
**Sum DAD1A**	204.7
**Sum DAD1C**	45.9
**Sum DAD1F**	1109.6

Supplementary Figure 7A. HPLC-DAD chromatogram for Vero E6 lysates

B

Qualitative - Detailed Report

Agilent

Peak Details (Area Percent at least 1%)

Signal Description: DAD1A,Sig=320,4 Ref=off *Spectra overlay at peak apex, up and down slopes*

Peak RT: 2.491 min **Area %:** 27.50%

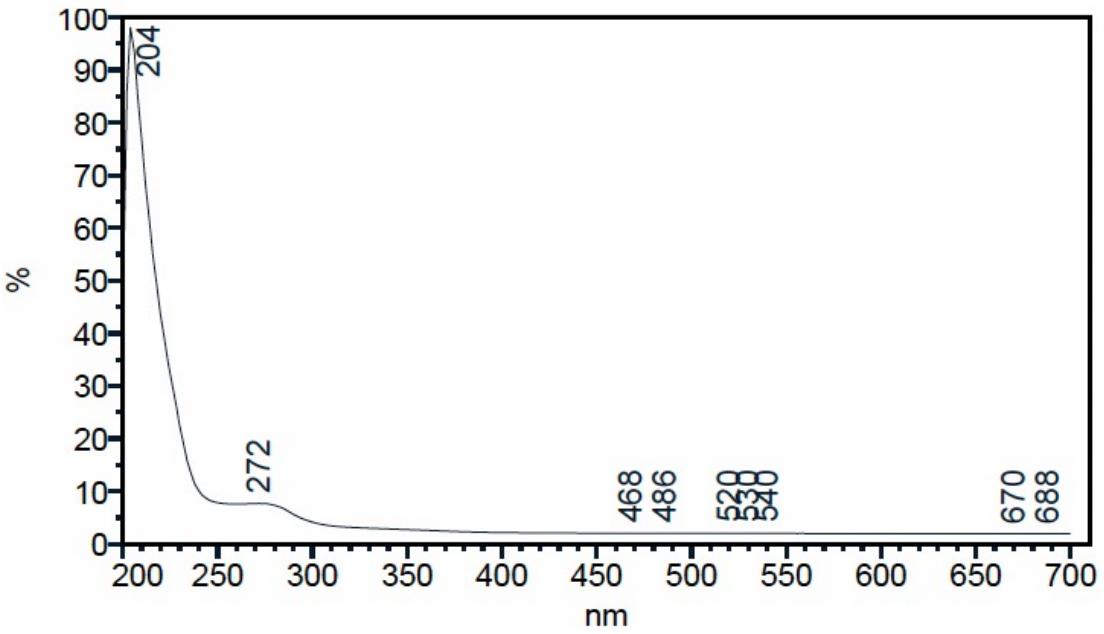

Peak RT: 2.611 min **Area %:** 9.33%

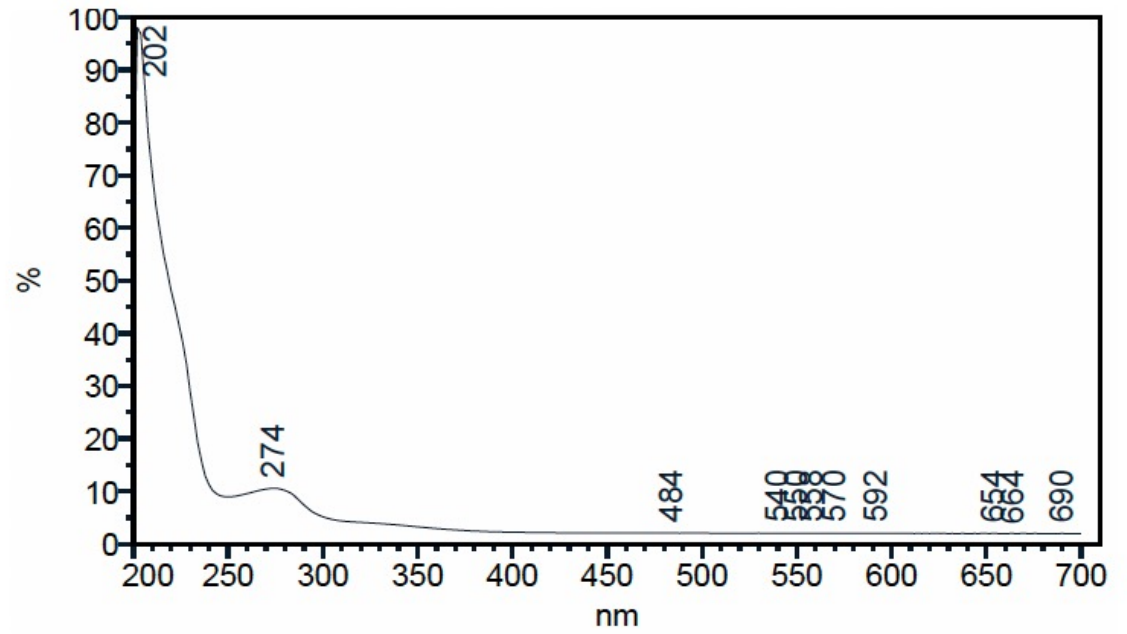

Peak RT: 2.808 min **Area %:** 51.82%

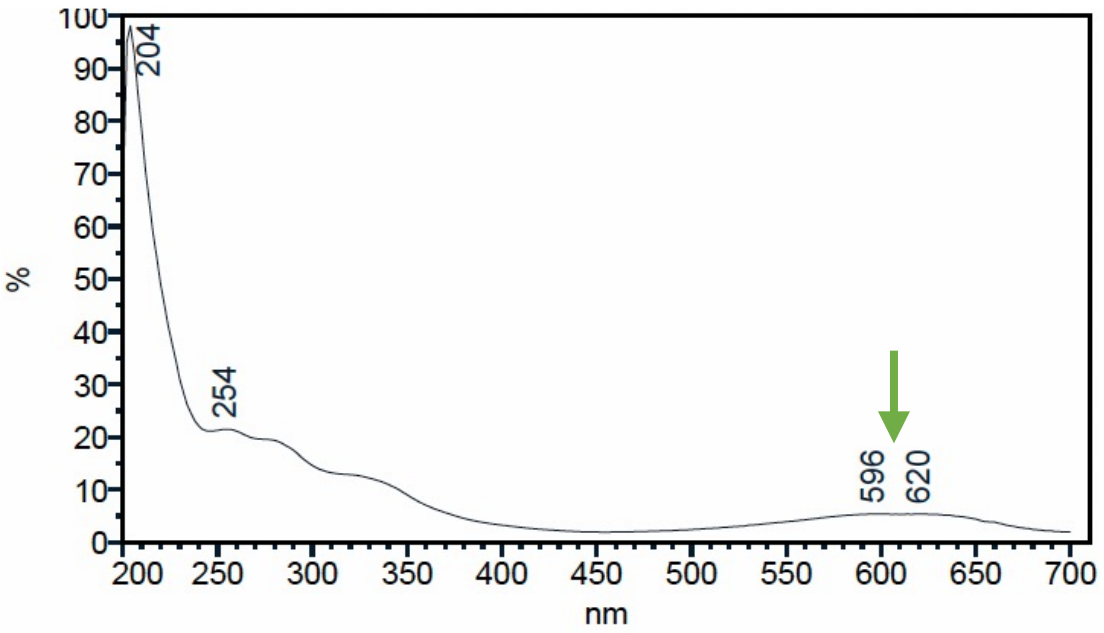

Peak RT: 3.964 min **Area %:** 5.17%

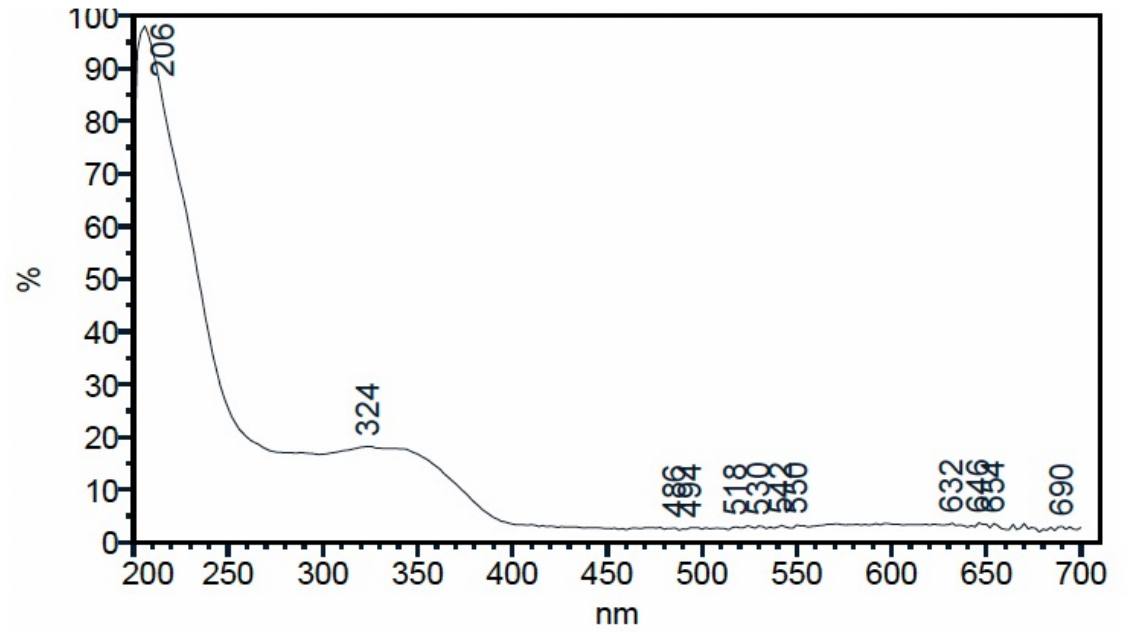

Peak RT: 5.064 min **Area %:** 6.18%

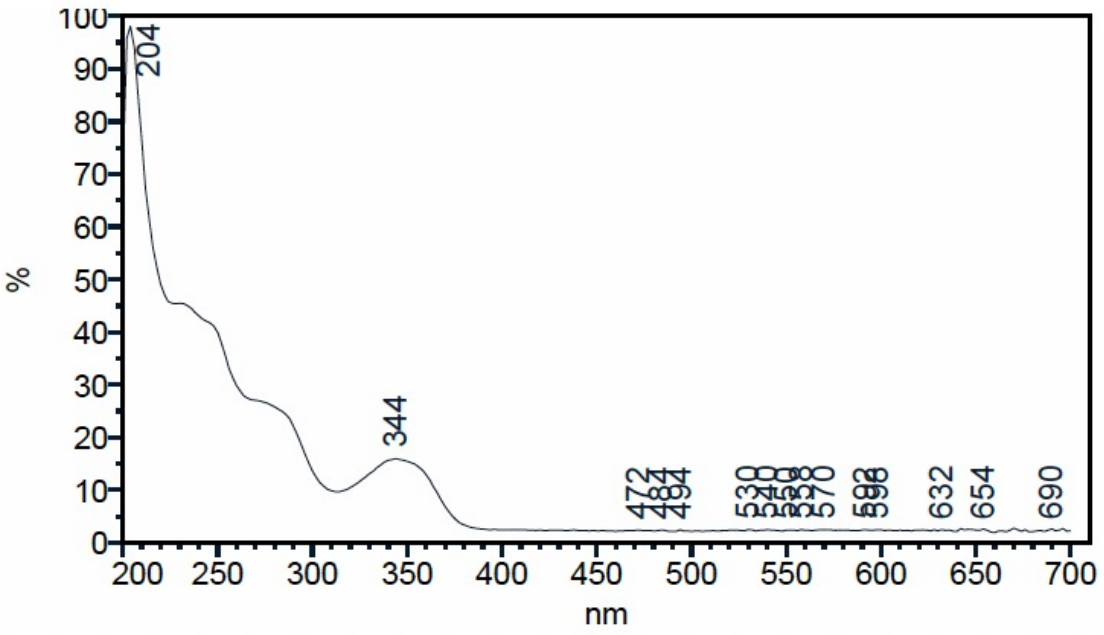

Supplementary Figure 7B. Peak Details of HPLC-DAD chromatogram for Vero E6 lysates, green arrow indicates pick of Reactive Blue 2.

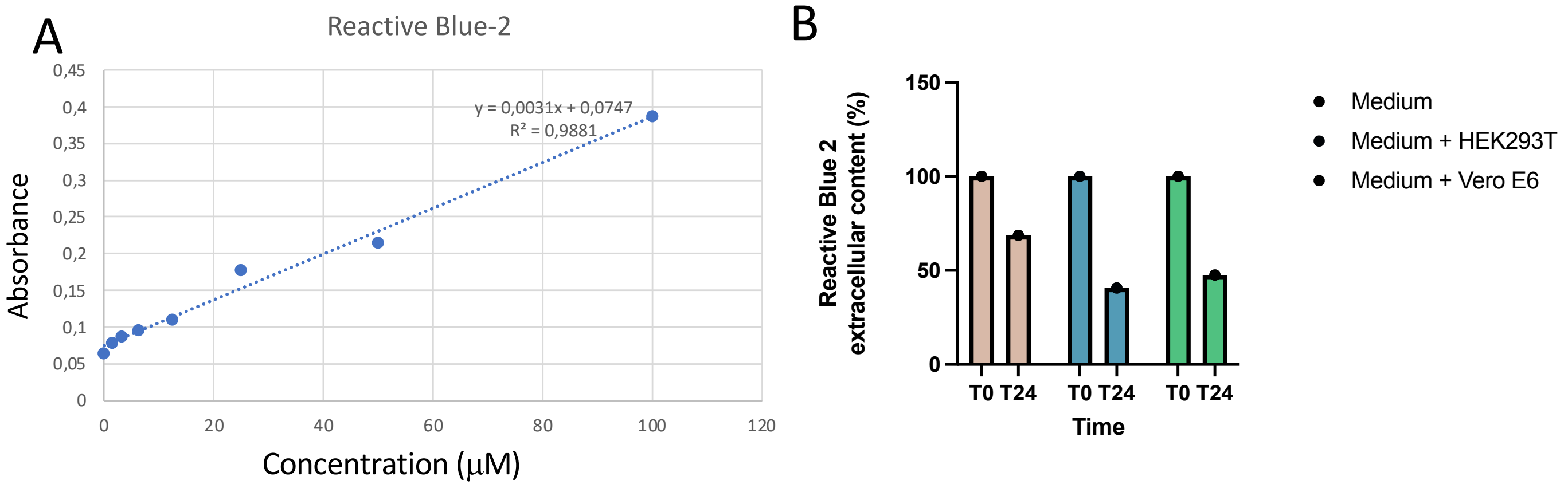

Supplementary Figure 8. (A) Calibration curve of Reactive Blue 2; (**B**) Reactive Blue 2 extracellular content in time 0 (T0) and time 24 hours (T24) post-treatment, in medium alone, medium with growing HEK293T and Vero E6 cells. Data are expressed in percentages with 100% representing T0 absorbance.

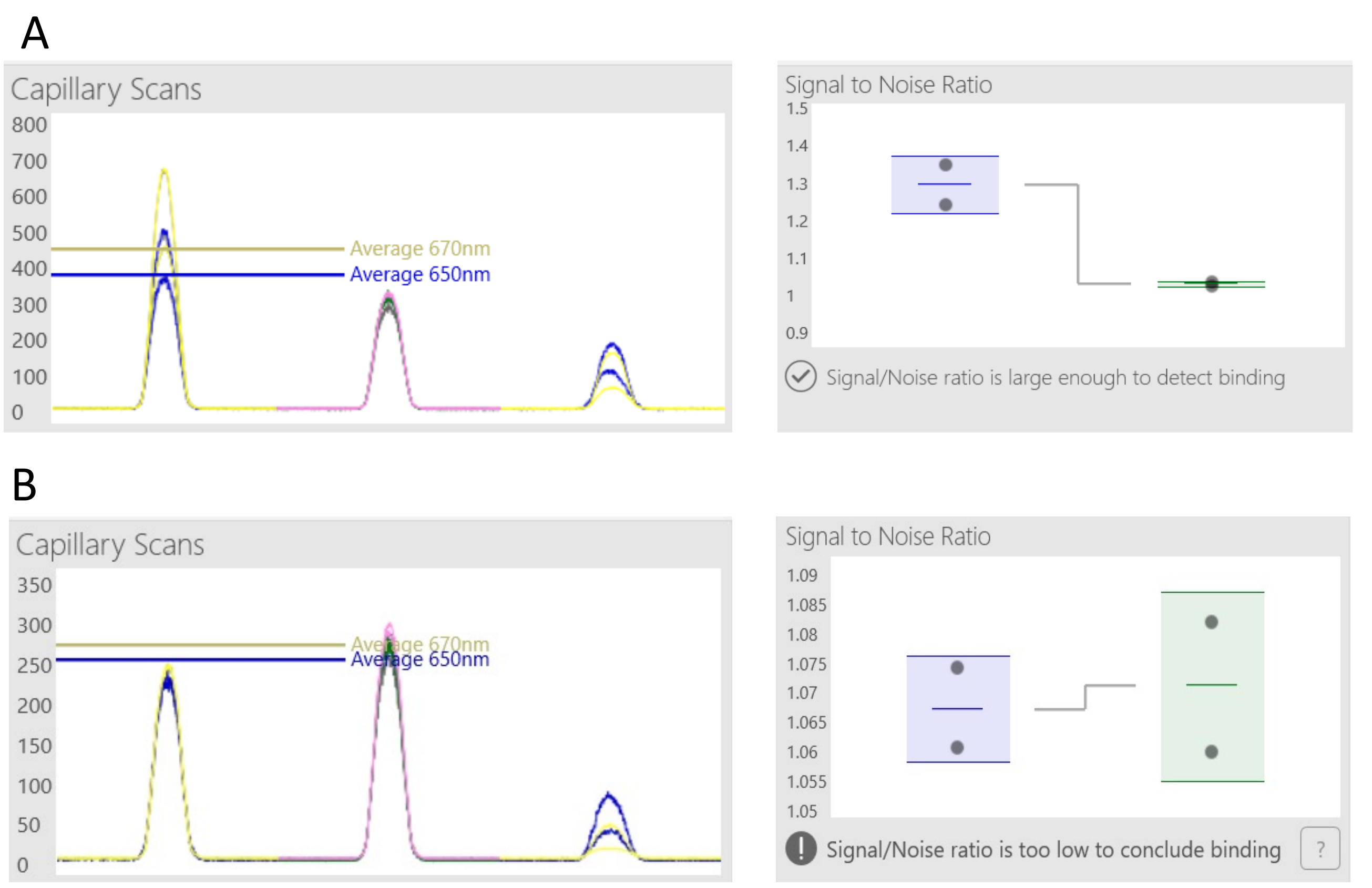

Supplementary Figure 9. Capillary scan and binding check results obtained from the reverse MST of Reactive Blue 2 and (**A**) Nsp15; or (**B**) RNA substrate.

Supplementary Methods

HPLC analysis

HPLC analysis were performed to confirm presence of Reactive Blue 2 in cellular lysates. We adopted a previously established protocol described in Claes *et al*., 2004. Briefly, HEK293T and Vero E6 cells were treated with Reactive Blue 2 for 24 h. Next day, cells at density of 10^5 cells/cm² were washed four times with PBS, scraped, and pelleted by centrifugation. The resulting pellet was resuspended in 50 mM Tris-HCl (pH 7.4). The cell suspension was then boiled for 5 minutes to denature proteins, followed by incubation with 5 U DNase for 1 hour at 37 °C. Next, 20 µg of proteinase K was added, and the mixture was incubated for 2 hours at 55 °C. Cellular lysates were treated with 0.5 ml of a solution constituted by ACN/H_2O/TFA (55:45:0.5) and subjected to a solid phase extraction (SPE) using a C-18 cartridge. The eluates were concentrated under reduced pressure and the residues, dissolved in 0.5 ml of ACN/H_2O/TFA (55:45:0.5), injected in a HPLC (Agilent 1260 Infinity II) equipped with a Diode Array Detector (DAD). The column used for HPLC analysis was a Kinetex C18 (i.d. 4.6 mm, 250 mm, 5 µm, Phenomenex); flow: 0.8 mL/min; mobile phase: ACN/H_2O/TFA (55:45:0.5). The reference compound and Vero and HEK cells lysates were monitored using the DAD settled at 260, 320, 392, and 620 nm. The injection volume was 30 µL.

Calibration curve of Reactive Blue 2 and measurement of adsorbance.

To measure a decrease in Reactive Blue 2 content in the extracellular media, absorbance at 595 nm was determined in the media alone and in the media of growing cells at Time 0 (T0) and after 24 hours (T24) of treatment. A calibration curve was generated to convert between concentration and absorbance (Supplementary Figure 8A), and differences in Reactive Blue 2 content in solution was expressed as percentages with 100% representing T0 absorbance. Absorbance was measured using a Victor Nivo microplate reader.
