# Supplementary material for: Identification of Nsp15 inhibitors restoring interferon-dependent antiviral activity against SARS-CoV-2
Source: Antiviral Res. Author manuscript; Available in PMC 2026 Sep 8. (PMC13551574; doi:10.1016/j.antiviral.2026.106443)
Supplement: Supplementary file 2 [file NIHMS2206332-supplement-Supplementary_table__Maloccu_et_al_2026.pdf]

Supplementary Table

**Omicron Nsp15**	MSLENVAFNVVNKGHFDGQQGEVPVSIINNTVYTKVDGVDVELFENKTTLPVNVAFELWAKRNIKPVPEVKILNNLGVD<br>IAANTVIWDYKRDAPAHISTIGVCSMTDIAKKPIETICAPLTVFFDGRVDGQVDLFRNARNGVLITEGSVKGLQPSVGPKQ<br>ASLNGVTLIGEAVKTQFNYYKKVDGVVQQLPETYFTQSRNLQEFKPRSQMEIDFLELAMDEFIERYKLEGYAFEHIVYGDF<br>SHSQLGGLHLLIGLAKRFKESPFELEDFIPMDSTVKNYFITDAQTGSSKCVCSVIDLLLDDFVEIIKSQDLSVVSKVVKVTIDY<br>TEISFMLWCKDGHVETFYPKLQ*
**MERS Nsp15**	MGLENIAFNVVKQGHFIGVEGELPVAVVNDKIFTKSGVNDICMFENKTTLPTNIAFELYAKRAVRSHPDFKLLHNLQADIC<br>YKFVLWDYERSNIYGTATIGVCKYTDIDVNSALNICFDIRDNCSLEKFMSTPNAIFISDRKIKKYPCMVGPDYAYFNGAIIRD<br>SDVVKQPVKFYLYKKVNNEFIDPTECIYTQSRSCSDFLPLSDMEKDFLSFDSDVFIKKYGLENYAFEHVVYGDFSHTTLGGL<br>HLLIGLYKRQQEGHIIMEEMLKGSSTIHNYFITETNTAAFKAVCSVIDLKLDDFVMILKSQDLGVVSKVVKVPIDLTMIEFM<br>LWCKDGQVQTFYPRLQ*

Supplementary Table 1. Sequences of Omicron Nsp15 and MERS Nsp15
